# 5-*N*-Arylaminothiazoles with pyridyl groups and their first-row transition metal complexes: synthesis, photophysical properties, and Zn sensing†

**DOI:** 10.1039/d2ra01694j

**Published:** 2022-05-16

**Authors:** Khurnia Krisna Puji Pamungkas, Toshifumi Maruyama, Toshiaki Murai

**Affiliations:** Department of Chemistry and Biomolecular Science, Faculty of Engineering, Gifu University Yanagido Gifu 501-1193 Japan mtoshi@gifu-u.ac.jp

## Abstract

A series of 5-*N*-arylaminothiazoles were synthesized with facile diversity-oriented synthesis from readily available starting materials *via* the reaction of thioamide dianions and thioformamides. The introduction of various substituents at the 2-position of a thiazole ring (*i.e.*, 2-pyridyl, 4-methylpyridyl, and phenyl groups) and on the nitrogen atom (*i.e.*, *p*-tolyl and phenyl groups) significantly influenced the absorption and emission spectra of the isolated compounds. X-ray analysis confirmed that the substituents at the amino site were twisted from a thiazole ring, while the formation of its nickel complex showed dinuclear metal complexes bridged with chlorine atoms. Moreover, the formation of zinc–thiazole complexes showed enhanced emission properties in solution and noticeable emission in a solid state. In addition, thiazole-bridged dypyrromethene type ligands showed high selectivity toward Zn^+2^, which make them good candidates for zinc sensing.

## Abstract 2

A series of 5-*N*-arylaminothiazoles were used to form isolable nickel complexes, whose structure was disclosed by X-ray structure analysis, and were used for the detection of zinc halides in solutions with the enhancement of emission properties.

## Graphical abstract

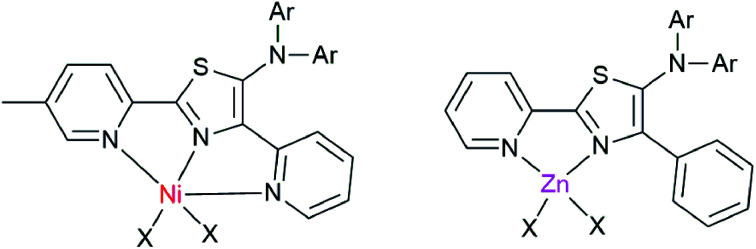

## Introduction

**Fig. 1:**
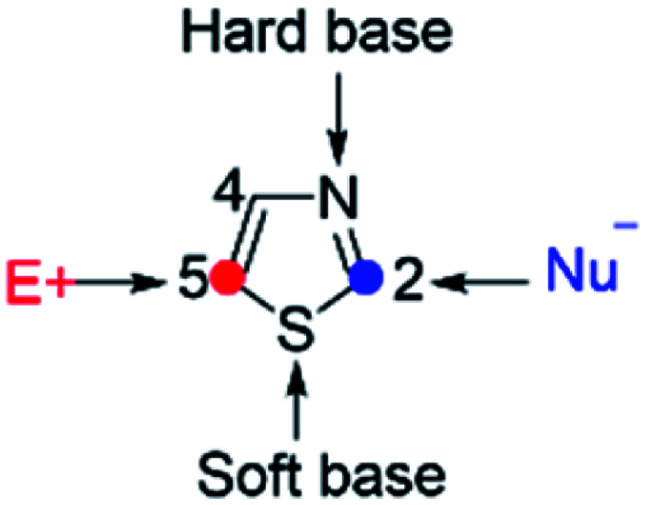
Thiazole structure.

Thiazoles are five-membered heteroaromatics that contain sulfur and nitrogen atoms in a cyclic ring system (Fig. 1). One of the distinctive features of thiazoles is that they have a sulfur atom, which is a soft base, and a nitrogen atom, which is a hard base. This implies that thiazoles can coordinate to a range of soft and hard metals. Moreover, the calculated π-electron densities of thiazoles suggest that C_5_ is the most favorable site for electrophilic substitutions, followed by C_4_, while C_2_ is a preferable site for nucleophilic substitutions.^1^

**Fig. 2:**
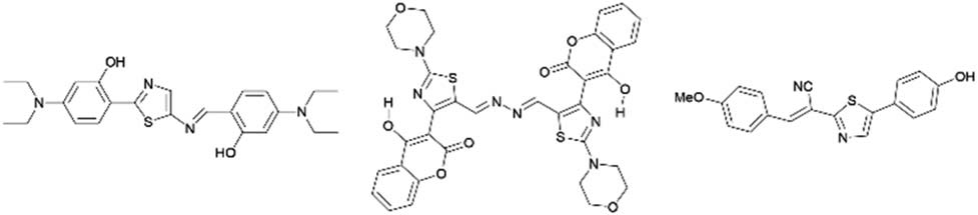
Selected examples of thiazole fluorescent compounds.

Many thiazole-based compounds are now in clinical use, such as the anticancer drug dasatinib,^2^ and the anti-HIV drug ritonavir.^3^ Additionally, thiazoles have three carbon atoms at the 2-, 4-, and 5-positions, to which a wide variety of substituents can be principally introduced. Therefore, a wide range of thiazole derivatives have been developed. On the other hand, the chemistry of thiazoles has been extended to the development of thiazole-based fluorescent materials. These fluorescent compounds exhibit unique photophysical properties such as aggregation induced emission (AIE),^4,5^ thermochromism,^4^ mechanochromism,^6^ and large Stokes shifts,^7^ and some of them are acid-responsive fluorescent compounds (Fig. 2).^8^

Thiazole can connect to several electron donor-containing fragments which exhibit coordination ability such as a pyridyl group. Due to this modification, several thiazoles coordinated to a boron atom have been reported to show acid-responsive properties^9^ and aggregation induced emission (AIE) with high quantum yields of up to 95% in the solid state.^10^ Moreover, thiazoles are also suitable as organic ligands to various metal. In fact, the presence of a metal surface closest to the fluorescent molecule can manipulate the decay rate of the fluorophore and produce drastic spectral changes.^11^ Moreover, interactions between a metallic surface with fluorophores have a beneficial effect on photophysical properties such as an increased quantum yield, improved photostability and a reduced lifetime of fluorophores.^12^

**Fig. 3:**
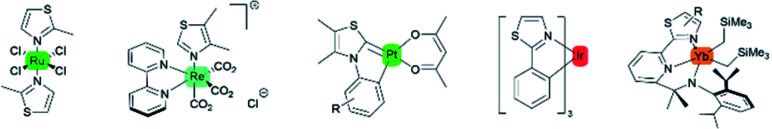
Selected examples of transition metal–thiazole complexes.

Intense studies on thiazole-based ligands coordinated to a transition metal have increased over the past several years. A considerable number of metal–thiazole complexes have been discovered to be catalytically^13,14^ and biologically active compounds for medical applications.^15,16^ In addition, the most commonly reported thiazoles coordinated to transition metals that show luminescence properties are complexes with ruthenium,^17–19^ iridium,^20^ rhenium,^21^ platinum,^22^ and lanthanoid elements (Fig. 3).^23–25^

**Fig. 4:**
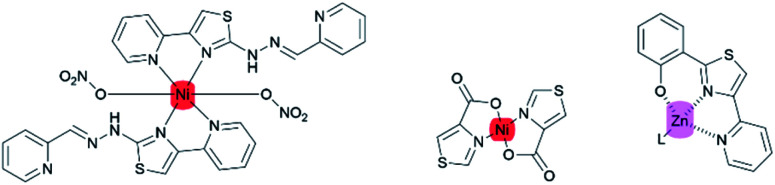
First-transition thiazole metal complexes.

Those works have mainly focused on the second- and third-row transition metals, while the uses of the first-row transition metals (Fe, Co, Ni, Co, and Zn) are less favorable due to their exceptionally short CT lifetimes arising from deactivation through low-lying ligand-field excited states.^26^ Nevertheless, the coexistence of nitrogen and sulfur atoms in a thiazole ring could promote the stabilization of Ni(ii) complexes.^27^ Some of the first-row transition metals such as nickel and zinc are widely used as complexing agents for various purposes. For example, zinc metal complexes with thiazole pyridine-based ligands are biologically active as anti-microbial and anti-tumor agents.^16^ Meanwhile, nickel coordinated to thiazole-4-carboxylic acid exhibited polymorphism properties.^28^ However, there have been only a few reports on first-row transition metal–thiazole complexes.^29,30^ (Fig. 4).

**Fig. 5:**
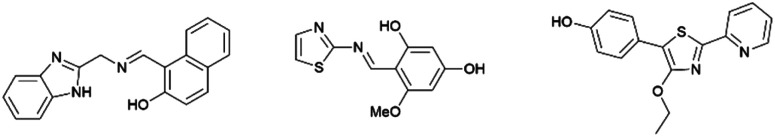
Fluorescent molecules for zinc sensing.

Recently, several fluorescent molecules have been used for the molecular recognition of zinc ions. However, due to its closed shell electron configuration (3d^10^), zinc ion is silent with respect to spectroscopic or magnetic signals.^31,32^ Therefore, a reliable technique for the detection of zinc ion is highly desirable. The most promising method for detecting zinc ion is by using fluorescence. Besides, fluorescence detection is fast, efficient, and simple.^33,34^ For example, imidazole-based compounds showed an increase in emission intensity in the presence of zinc ions.^35^ Meanwhile, zinc sensing based on thiazole compounds showed turn on–off emission properties^36^ and a strong emission response and a high quantum yield on addition of zinc ions (Fig. 5).^37^

Our group has been interested in the chemistry of 5-*N*-arylaminothizoles for many years. Therefore, a considerable number of these compounds have been developed.^38–44^ 5-*N*-Arylaminothiazoles are a class of fluorescent compounds bearing a flexible conformational geometry with non-planar carbon skeletons.^38^ These compounds are synthesized by reacting secondary thioamide and thioformamides *via* thioamide dianions and thiazolines. This synthetic protocol allows us to access a wide range of unprecedented 5-*N*-arylaminothiazoles with various substituents at the 2- and 4-positions of a thiazole ring.

Herein we report a series of 5-*N*-arylaminothiazole having pyridyl groups and their first-row transition metal complexes. The synthesis, structural elucidation, and photophysical properties both in a solid state and in solution are discussed.

## Results and discussion

### Synthesis of 5-aminothiazolines and 5-*N*-arylaminothiazoles

**Scheme 1:**
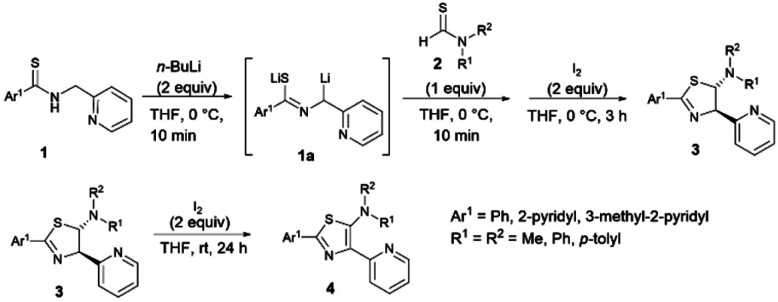
Synthesis of 5-aminothiazolines and 5-*N*-arylaminothiazoles.

The synthesis of ligands is depicted in Scheme 1. The new thiazole ligands were synthesized by deprotonation of secondary thioamides 1 with 2 equivalents of organolithium (*n*-BuLi) at low temperature to generate thioamide dianions 1a. Addition of thioformamides 2 and iodine to the reaction mixture gave 5-aminothiazolines 3 as major products.^44^ Further oxidation of 5-aminothiazolines 3 with 2 equivalents of iodine resulted in the formation of the corresponding 5-aminothiazole ligand 4 in low to good yields. Following this reaction, a wide range of substituents possessing electron-donating and -withdrawing properties could be introduced to the 2-, 4-, and 5- positions of the thiazole ring.

**Fig. 6:**
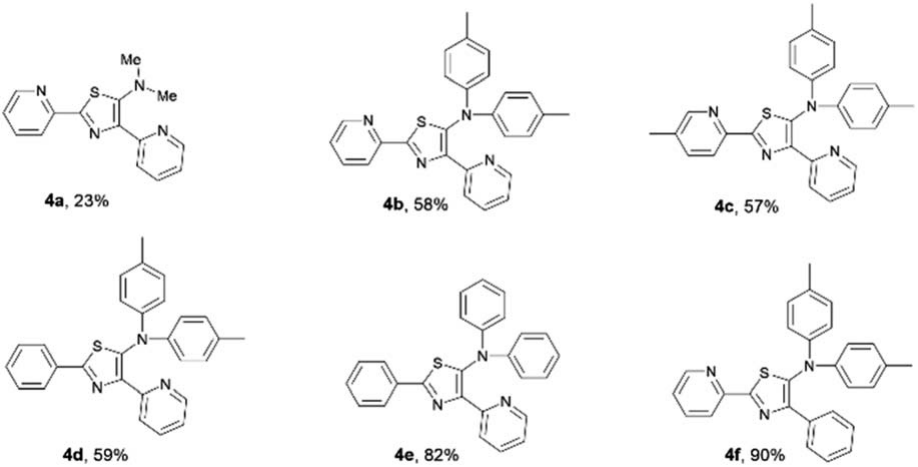
A series of isolated 5-*N*-arylaminothiazoles.

As a result, the introduction of pyridyl groups at the 2- and 4-positions gave 4a in 23% yield. Replacement of a methyl group with other functional groups at the 5-position of a thiazole ring led to an increase in the yield of the product. Incorporation of a tolyl group significantly improved the yield of the product 4b to 58%. In addition, the introduction of a methyl group at the *para* position of a pyridyl moiety gave 4c in 57% yield. Furthermore, the incorporation of pyridyl groups only at the 2- or 4-positions of a thiazole ring afforded thiazole products 4d, 4e, and 4f in yields of 59%, 82% and 90%, respectively (Fig. 6).

**Scheme 2:**
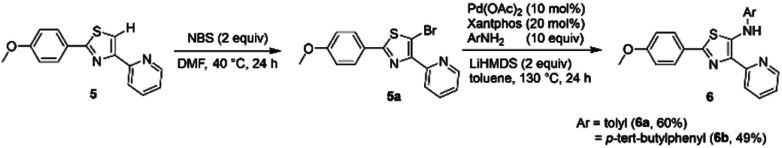
Synthesis of dipyrromethene-type ligands 6.

In addition, we also synthesized dipyrromethene-type ligands 6 following the protocol reported previously by our group.^9^ Initially, the 5-H thiazoles 5 undergo bromination reaction followed by Buchwald–Hartwig amination to give the corresponding thiazole ligands 6a and 6b in yields of 60% and 49%, respectively (Scheme 2).

### Synthesis of first-row transition metal–thiazole complexes

**Fig. 7:**
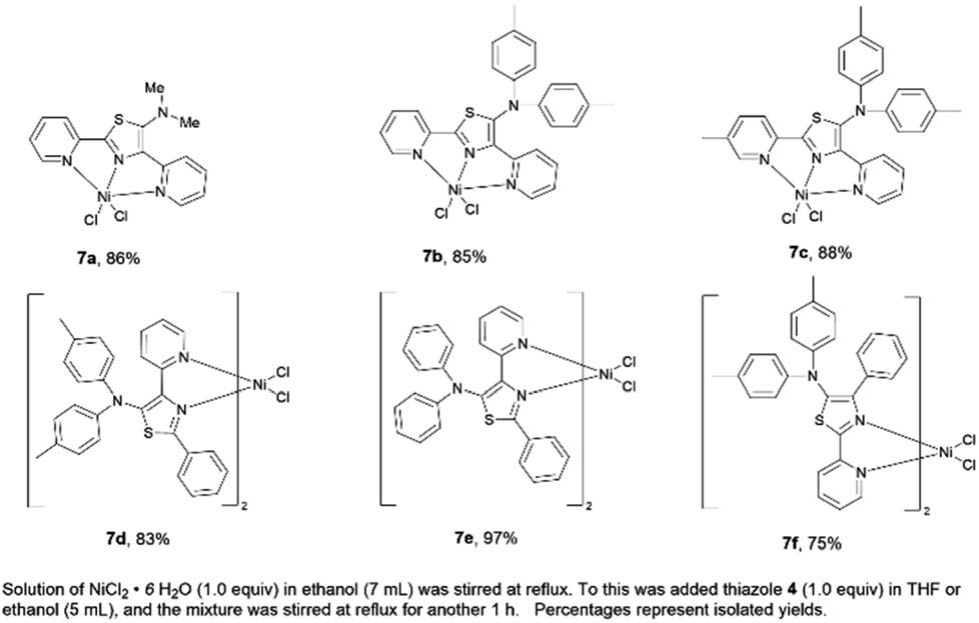
A series of isolated nickel–thiazole complexes.

A series of nickel–thiazole complexes 7 were prepared in two steps: (i) reflux of the metal source in ethanol under an inert atmosphere, (ii) synthesis of the desired final products by reacting the corresponding ligand 4 with nickel chlorides under reflux. As a result, all of the metal complexes were isolated in moderate to high yields (Fig. 7). ESI-mass analysis suggested two sets of nickel complex formations such as [Ni(L)X_2_] for nickel complex having a tridentate ligand (7a, 7b, and 7c) and [Ni(L_2_)X_2_] for nickel complex having a bidentate ligand (7d, 7e, and 7f).

**Fig. 8:**
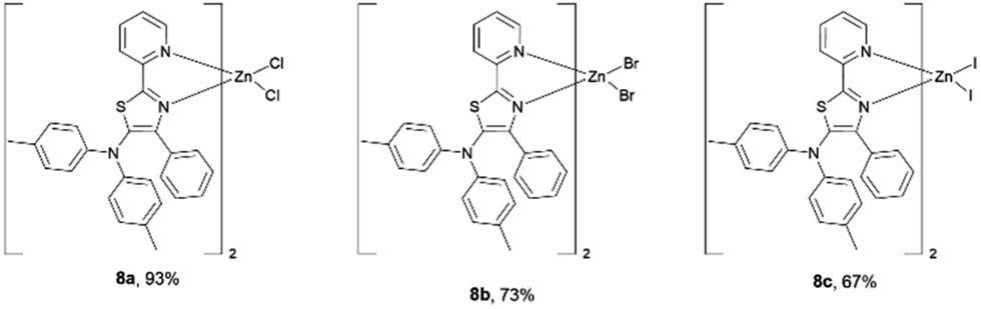
A series of isolated zinc–thiazole complexes.

Having succeeded in the synthesis of nickel–thiazole complexes, we then turned our attention to the synthesis of metal–thiazole complexes using other first-row transition metal atoms such as zinc. A series of zinc–thiazole metal complexes were synthesized by simply mixing the corresponding thiazoles 4f with zinc halides at room temperature. Following this procedure, new zinc thiazole complexes 7 with different halides were synthesized in moderate to high yields (Fig. 8). To our delight, these complexes showed good solubility in most organic solvents. ESI-mass analysis suggested that the zinc atom is coordinated to two bidentate thiazole ligands to form 2 : 1 metal complexes [Zn(L_2_)X_2_].^44^

**Fig. 9:**
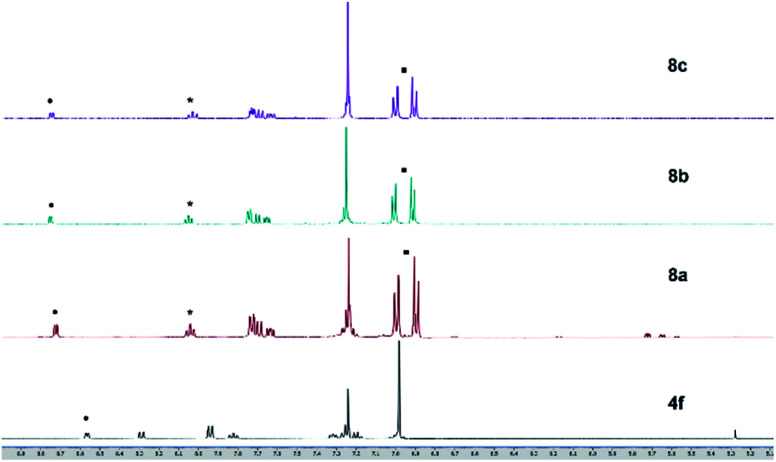
^1^H NMR (500 MHz, CDCl_3_) charts of ligand 4f and zinc thiazole complexes 6a–c.

Solutions of these zinc complexes in deuterated chloroform were prepared for NMR analyses. With the addition of 1 equivalent of zinc ion, the signals of protons of the thiazole ligand at 8.57 ppm and 7.83 ppm were shifted downfield to at 8.75 ppm and 8.04 ppm, respectively. Furthermore, in the formation of zinc complexes, two new doublet peaks integrated for 8 protons assigned to phenyl rings in the tolyl groups appeared at 7.00 ppm and 6.90 ppm, respectively. However, the proton signal and chemical shift of the complexes are almost identical despite having different halogens at the zinc center (Fig. 9). These results indicate the zinc–thiazole complexes 8 are formed on the addition of zinc halides.

### X-ray crystallography analyses

**Fig. 10:**
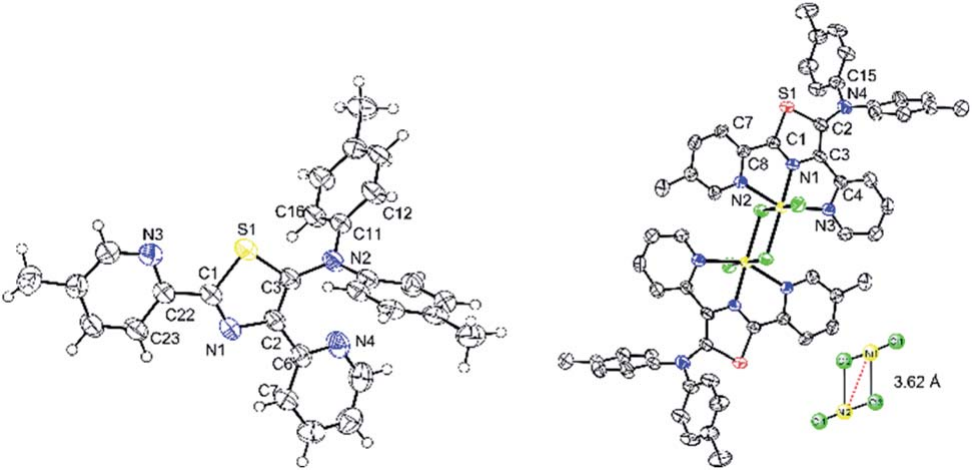
X-ray structure of thiazole 4c (left) and nickel–thiazole complex 7c (right). Hydrogen atoms are omitted for clarity.

High-quality single green crystals of thiazoles 4c were isolated in the triclinic space group *P*1̄ (Fig. 10). X-ray crystallography of 4c confirmed the twisted conformation geometry of amino groups at the 5-position of a thiazole ring with torsion of 108.0° (S1–C3–N2–C11). Meanwhile, two pyridyl groups attached at the thiazole ring were located almost in the same plane as the thiazole ring. Moreover, a green crystal of 7c showed the formation of dinuclear nickel complexes with a *trans* configuration in a distorted octahedral geometry (Fig. 9). Two bridging Cl atoms connect two central nickel atoms between two dimers with a distance of 3.620 Å between Ni1–Ni2 (inset: Fig. 10). The distance of bridging Ni1–Cl2 was 2.600 Å, which is longer than that of Ni1–Cl3 2.363 Å. The atoms Cl1–Ni1–Cl2 were oriented almost linearly with a bond angle of 177.55 (4)°, while the atoms Cl2–Ni1–N2 were oriented almost perpendicularly with a bond angle of 89.15°. The torsion angle of the amino groups in complex 7c (S1–C2–N4–C15) were 43.31°. Moreover, the distances of Ni1–N1 (thiazole ring) and Ni1–N3 (pyridyl group) was found to be 1.970 and 2.198 Å, respectively.

### Photophysical properties of 5-*N*-arylaminothiazoles and their complexes in solution

**Fig. 11:**
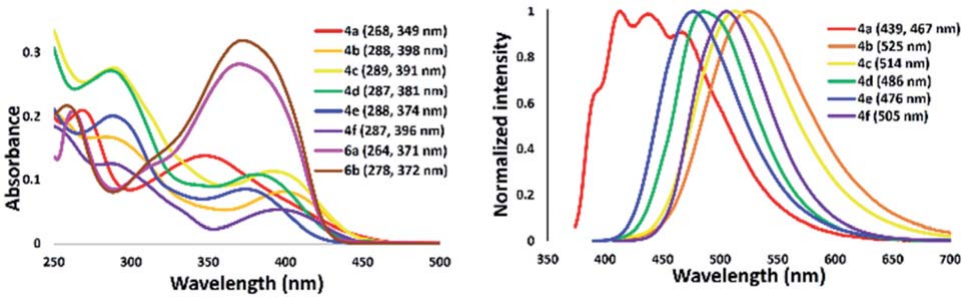
Absorption (right) and emission spectra of 5-*N*-arylaminothiazoles in chloroform [10^−5^ M].

The absorption and emission spectra of isolated 5-aminothiazole derivatives in chloroform are shown in Fig. 11 and Table 1. The absorption maxima range from 349 to 398 nm. That of thiazole 4a was in the shortest absorption region, and gradually shifted to longer wavelengths with introduction of a tolyl group to the nitrogen atom at the 5-position of a thiazole ring, such as 4b. Interestingly, the incorporation of a methyl group at the *para* position of a pyridyl group slightly shifted the absorption maxima to a shorter wavelength, such as in 4c. Further blue-shift absorption was observed by replacing a tolyl group with a phenyl group at the 2-position of the thiazole ring of 4d. In addition, when a phenyl group was placed at the 2-position and the nitrogen atom at the 5-position of a thiazole ring for 4e, the absorption maxima shifted to 374 nm, while the introduction of a phenyl group at the 4-position of a thiazole ring for 4f shifted the absorption maxima to 396 nm. Furthermore, the emission maxima of 5-*N*-arylaminothiazoles varied from 467–525 nm depending on the substituents. These results showed that the photophysical properties of 5-*N*-arylaminothiazoles could be finely tuned simply by introducing different substituents at the thiazole ring. In addition, the emission maxima of 6a and 6b were observed at 371 nm and 372 nm, respectively. However, these dipyrromethene-type ligands did not show emission properties.

**Table 1:** Spectroscopic data of a series of 5-*N*-arylaminothiazoles^a^

Thiazole	*λ*_abs_ (nm)	log *ε*	*λ*_ex_ (nm)	*λ*_em_ b (nm)	*ν*_ss_ [cm^−1^] (nm)	*φ*_F_ c
4a	349	4.14	359	467	[7240] (118)	0.04
4b	398	3.90	397	525	[60781] (127)	0.13
4c	391	4.05	395	514	[61201] (123)	0.16
4d	381	4.03	377	486	[56701] (113)	0.2
4e	374	3.92	376	476	[5729] (102)	0.31
4f	396	3.72	392	505	[5450] (109)	0.42
6a	371	4.44	—	—	—	—
6b	372	4.50	—	—	—	—

a In chloroform [conc. = 10^−5^ M].

b Excited in *λ*_max_.

c Absolute quantum yield of fluorescence.

**Fig. 12:**
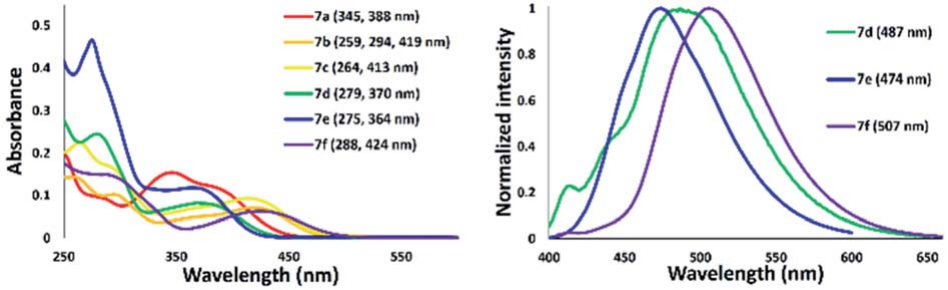
Absorption (right) and emission spectra of nickel–thiazole complexes in chloroform [10^−5^ M].

We next examined the spectroscopic properties of nickel–thiazole complexes 7 in dilute chloroform (Fig. 12 and Table 2). The longest absorption spectra of nickel complexes 5 ranged from 278 to 424 nm. The absorption maxima of 7a were observed at 388 nm and were significantly red-shifted for a thiazole complex having a tolyl group on the nitrogen atom at the 5-position of a thiazole ring such as 7b. The presence of a methyl group at the *para* position of the pyridyl group slightly shifted the absorption maxima to a shorter wavelength of 7c. The absorption maxima of 7d were blue-shifted to 370 nm with the introduction of a phenyl substituent at the 2-position of a thiazole ring. Meanwhile, replacement of a tolyl group by adding a phenyl group at the 2-position and on the nitrogen atom attached to a thiazole ring shifted the absorption maxima to 364 nm, such as in 7e. Moreover, a nickel–thiazole complex having a phenyl group at the 4-position instead of pyridyl and tolyl groups on the nitrogen atom 7f was observed at 424 nm. In addition, the formation of a nickel–thiazole complex turned the emission properties off, as in 7a, 7b, and 7c. No emission was observed in either in a solid state or in solution. In contrast, the formation of nickel complexes 7d, 7e and 7f had no effect on emission, and they are still emissive in a solution to some extent under UV illumination.

**Table 2:** Spectroscopic data of nickel–thiazole complexes^a^

Thiazole	*λ*_abs_ (nm)	log *ε*	*λ*_ex_ (nm)	*λ*_em_ b (nm)	*ν*_ss_ [cm^−1^] (nm)	*φ*_F_ c
7a	388	4.06	359	—	—	—
7b	419	3.85	397	—	—	—
7c	413	3 96	395	—	—	—
7d	370	3.91	377	487	[6493] (117)	0.04
7e	364	4.07	376	474	[6375] (110)	0.03
7f	424	3.79	392	507	[3861] (83)	0.02

a In chloroform [conc. = 10^−5^ M].

b Excited in *λ*_max_.

c Absolute quantum yield of fluorescence.

**Fig. 13:**
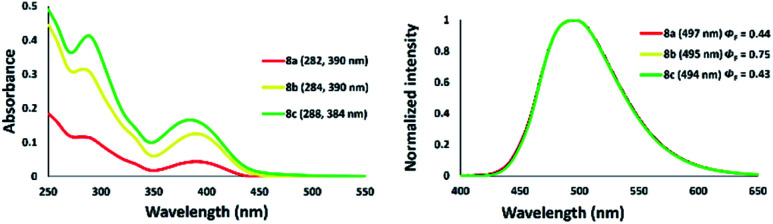
Absorption (right) and emission spectra of zinc–thiazole complexes 8a–c in THF [10^−5^ M].

The photophysical properties of a series of zinc–thiazole complexes investigated in a THF solution are shown in Fig. 13. The absorption maxima of zinc complexes 8a–c in THF were observed at 390 nm in all cases, while that of zinc thiazole having an iodine 8c was slightly blue-shifted to 384 nm. Meanwhile, their emission maxima showed no significant change even when we introduced different halogens on the zinc center. Interestingly, zinc–thiazole complex containing a bromide 8b showed a high quantum yield of up to 75%. Fluorescence lifetime decay of thiazole ligand 4f and zinc-complexes 8 in THF was in the range 4.43–4.94 ns. The thiazole 4f exhibited fluorescence lifetimes about 4.93 ns. Introduction of dichloride and zinc dibromide to the zinc center shortened the fluorescence lifetime such as 8a and 8b. Interestingly, zinc–thiazole complex having an iodine atom in the metal center 8c possessed the longest average fluorescence lifetimes (Table 3).

**Table 3:** Spectroscopic data of 5-*N*-arylaminothiazole 4f and zinc–thiazole complexes in THF [10^−5^ M]^a^

Thiazole	*λ*_abs_ (nm)	log *ε*	*λ*_ex_ (nm)	*λ*_em_ b (nm)	*ν*_ss_ [cm^−1^] (nm)	*φ*_F_ c	*χ* ^2^ d	*τ* d (ns)
4f	390	3.92	385	495	[5504] (105)	0.5	1.8	4.93
8a	390	3.64	384	497	[5520] (107)	0.4	2.7	4.86
8b	390	4.1	384	495	[5504] (105)	0.8	2.1	4.43
8c	384	4.22	386	494	[5798] (110)	0.4	2	4.94

a Measured in THF.

b Excited in *λ*_max_.

c Absolute quantum yield of fluorescence.

d Excited at 365 nm.

### Solvatochromism properties of 5-*N*-arylaminothiazoles and their complexes

**Fig. 14:**
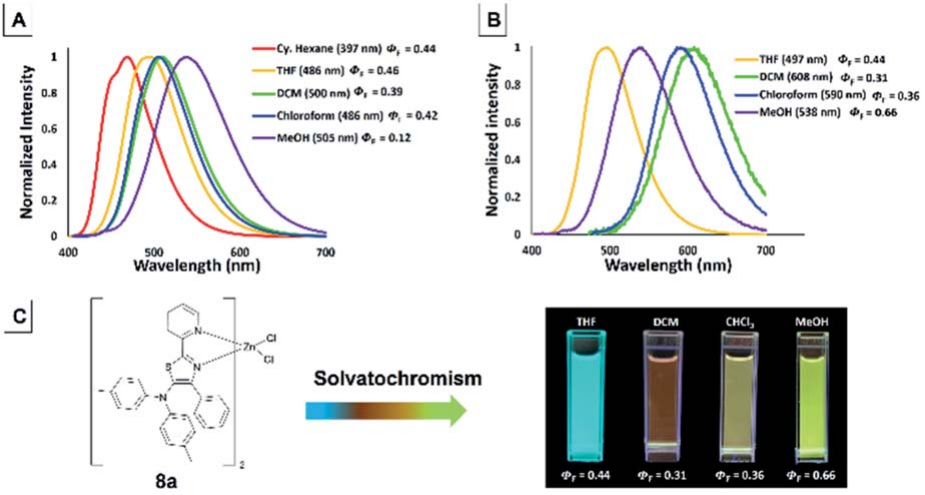
Emission spectra of 5-aminothiazole 4f (A) and zinc–thiazole complex 8a (B) [10^−5^ M]. Change in the color of emission for 8a in solvent with different polarities (C).

The emission color of the isolated 5-*N*-arylaminothiazoles varied with the solution polarity exhibited a tunable emission wavelength in the range of 397–505 nm.^44^ The emission maxima of 4f strongly depended on the solvent polarity, and thus their peak emissions were greatly affected. Large red shifts in the emission maxima (Fig. 14) were observed upon changing from a nonpolar (cyclohexane) to a polar (MeOH) solvent and cover blue to green color with low quantum yield. In addition, zinc–thiazole complex 8a also showed solvatochromism properties and transitioned from a blue to green color. Uniquely, the emission maxima of 8a in halogenated solvents (dichloromethane and chloroform) were more red-shifted compared to its emission maxima in non-halogenated (THF and MeOH) solvents (Fig. 14). To our delight, zinc–thiazole complex 8a showed comparable quantum yields with the free ligand 4f.

### Photophysical properties of 5-*N*-arylaminothiazoles and their complexes in a solid state

In addition to showing bright emission in solution, 5-*N*-arylaminothiazoles 4 and their zinc–thiazole complexes 8 also exhibited emission in a solid state. However, thiazole 4a and the formation of nickel–thiazole complexes 7 did not show any noticeable emission properties in a solid state. Thiazoles 4b–f showed emission in a solid state with a quantum yield (*φ*_F_) reaching up to 52%. The absorption maxima of 4b–d were observed in almost the same absorption region, while of those 4e and 4f were slightly blue-shifted to 427 nm and 404 nm, respectively. Their longest emission maxima appeared from 469 nm to 542 nm with low to moderate quantum yields.

**Fig. 15:**
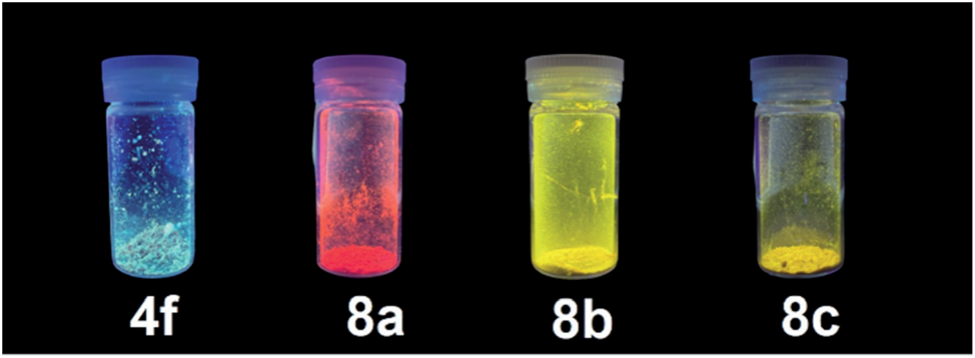
Emission color in a solid state of 5-aminothiazole 4f and zinc–thiazole complexes 8a–c under UV radiation at 365 nm.

The absorption and emission maxima of zinc–thiazole complexes 8 in a solid state were significantly different than those of the free ligands (Table 4). While the longest absorption maxima ranged from 440 nm to 452 nm, while their emission maxima varied depending on the halogens on the zinc center. The zinc thiazole complex with chlorine atoms at the zinc center 8a showed a significant red-shift to 611 nm compared to that of the free ligand 4f at 475 nm. Furthermore, the introduction of bromine (8b) and iodine atoms (8c) to the zinc center shifted the emission maxima to a shorter emission wavelength at 550 nm and 548 nm, respectively. The emission decay analysis showed significant decreasing of fluorescence lifetimes of zinc–thiazole complexes 8 compared to its free ligand 4f (Table 4). Moreover, the quantum yield of the complexes dropped significantly in a solid state. However, the difference in emission color under UV irradiation at 365 nm was still easily visible with the naked eye (Fig. 15).

**Table 4:** Spectroscopic data of 5-*N*-arylaminothiazoles and zinc–thiazole complexes in a solid state

Thiazole	*λ*_abs_ (nm)	*λ*_ex_ (nm)	*λ*_em_ a (nm)	*ν*_ss_ [cm^−1^] (nm)	*φ*_F_ b	*χ* ^2^ c	*τ* c (ns)
4b	442	439	495	[2411] (53)	0.21	1.66	2.53
4c	444	437	542	[3438] (80)	0.06	3.5	2.47
4d	444	437	475	[1469] (31)	0.21	1.56	2.77
4e	427	428	469	[2097] (42)	0.15	1.36	1.91
41	404	431	475	[3609] (71)	0.52	1.54	3.03
8a	440	370	611	[6360] (171)	0.02	3.21	121
8b	440	365	550	[4545] (110)	0.05	2.41	1.99
8c	452	492	548	[3875] (96)	0.02	2.66	0.23

a Excited in *λ*_max_.

b Absolute fluorescence of quantum yield.

c Excited at 365 nm.

### Use of 5-*N*-arylaminothiazoles as a fluorescence probe for detection of zinc ion

**Fig. 16:**
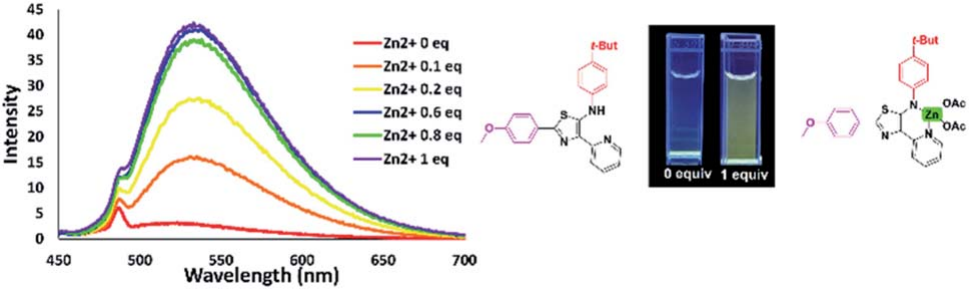
Fluorescence titration of 6b with different amounts of zinc ions in THF solution [10^−5^ M].

Coordination of Zn^2+^ with dipyrromethene-type ligands 6 turned on the emission properties for 6b. Considering this unexpected result, we then investigated the possibility of using 6b for Zn^2+^ sensing. We initially titrated of various metal cations such as Cu^2+^, Ca^2+^, Fe^3+^, and Zn^2+^ into a THF solution of dipyrromethene-type ligand 6b. As a result, compound 6b showed high selectivity for Zn^2+^ ions. With the addition of Zn^2+^, the emission intensity at 533 nm continued to increase with the appearance of a green emission with a low quantum yield (*φ*_F_) of 0.02 under UV irradiation at 365 nm (Fig. 16).

**Fig. 17:**
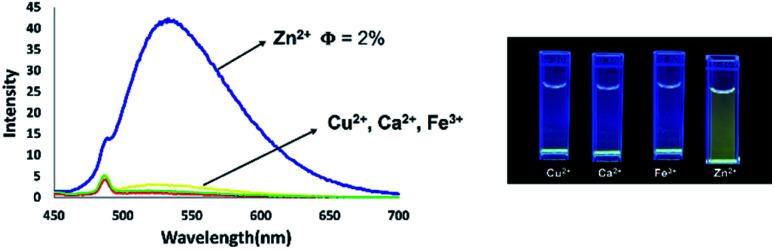
Fluorescence titration of 6b with different cations in THF solution [10^−5^ M].

In contrast, the emission properties of 6b towards cations other than Zn^2+^ did not show any change in emission intensity even after the addition of 1 equivalent of cation. These results imply that 6b has excellent potential as a candidate for Zn^2+^ fluorescence sensing (Fig. 17).

## Conclusions

In summary, a series of 5-*N*-arylaminothiazole derivatives having pyridyl groups were successfully synthesized and systematically characterized. The absorption and emission properties of isolated 5-*N*-arylaminothiazoles were finely tuned simply by varying the substituents attached to the thiazole ring and the nitrogen atom at the 5-position. The longest absorption spectra ranged from 349 to 396 nm. They also showed solvatochromism properties in solvent with different solvent polarities. X-ray analyses revealed that the isolated 5-*N*-arylaminothiazole adopts a highly twisted conformation, wherein the diarylamino group deviates from the thiazole ring. The crystal structure of nickel–thiazole complex 7c confirmed the formation of dinuclear metal complexes with chlorine atom acting as a bridge between two nickel complexes. However, the formation of nickel complexes turned off the emission properties of some complexes. In contrast, the formation of zinc–thiazole complexes showed emission both in solution and in a solid state. Moreover, dipyrromethene-type ligands showed consistent enhancement of emission intensity upon the addition of Zn^2+^. Therefore, these ligands can be used for zinc sensing. Further applications of 5-*N*-arylaminothiazoles as chemosensors and biosensors will be reported in due course.

## Experimental section

### General procedure A: preparation of thiazoles

To a solution of thiazolines (1 equiv.) in THF was added iodine (2 equiv.) at room temperature, and the mixture was stirred for 24 h. The resulting mixture was poured into a saturated aqueous solution of Na_2_S_2_O_3_ and extracted with Et_2_O. The organic layer was dried over MgSO_4_ and concentrated *in vacuo*. The residue was purified by column chromatography (SiO_2_) to give the corresponding thiazoles.

### General procedure B: preparation of dipyrromethene type ligands

Pd(OAc)_2_ (10 mol%) and xantphos (20 mol%) were weighted into Schlenk tube that was sealed with a septum and purged with argon (3 times). Toluene (6 mL) then injected, and the reaction was continued for 15 min at 130 °C. 5-Bromothiazole (1 equiv.) in 4 mL toluene was then injected *via* syringe followed by the amine (10 equiv.) and LiHMDS (2 equiv.). The reaction mixture was filtered through a bed of Celite and washed with CH_2_Cl_2_. The filtrate was concentrated *in vacuo* and purified *via* silica gel flash column chromatography (SiO_2_, hexane : EtOAc) to give the corresponding thiazoles.

### General procedure C: preparation of nickel thiazole complexes

NiCl_2_·6H_2_O (1 equiv.) was taken in ethanol and was heated to reflux. The 5-*N*-arylaminothiazoles (1 equiv.) in a EtOH solution was added dropwise to the refluxing solution and was further refluxed for another 1 h. The final mixture was concentrated *in vacuo*. The obtained participate product was then washed by ethanol and diethyl ether. To give pure nickel–thiazole complexes.

### General procedure D: preparation of zinc thiazole complexes

To a solution of thiazoles (1 equiv.) in THF was added zinc halides (1 equiv.) at room temperature, and the mixture was stirred for 24 h. The precipitate was then washed with hexane and Et_2_O to give pure zinc–thiazole complexes.

### Fluorescence lifetime analysis

All zinc complexes were analyzed in dilute THF [10^−5^ M] using the Fluorescence Lifetime Spectrometer Quantaurus-Tau® C16361 Series with a measurement time range of 50 ns and frequencies at 5 MHz.

### *N*,*N*-Dimethyl-2,4-di(pyridin-2-yl)thiazol-5-amine (4a)

#### General procedure A

4a (0.193 g, 23%) as a yellow solid (mp: 64–65 °C); IR (KBr) 3420, 2923, 1586, 1564, 1501, 1472, 1433, 1413, 1366, 1145, 1001, 783, 741 cm^−1^; ^1^H NMR (400 MHz, CDCl_3_) *δ* 2.94 (s, 6H) 7.21–7.22 (m, 1H) 7.23 (t, 1H) 7.74 (td, 1H) 7.79 (td, 1H) 8.12 (d, *J* = 8.24 Hz, 1H) 8.27 (d, *J* = 8.24 Hz, 1H) 8.53 (d, *J* = 4.58 Hz, 1H) 8.75 (d, 1H); ^13^C NMR (400 MHz, CDCl_3_) *δ* 46.41, 118.91, 121.13, 123.43, 123.51, 136.65, 136.79, 148.82, 149.21, 151.95, 154.11, 154.82, 157.13, 135.57; (EI) *m*/*z* 282 (M^+^); HRMS (EI) calcd for C_15_H_14_N_4_S, 282.0939; found, 282.0938.

### 2,4-Di(pyridin-2-yl)-*N*,*N*-di-*p*-tolylthiazol-5-amine (4b)

#### General procedure A

4b (0.23 g, 76%) as a greenish yellow solid (mp: 171–172 °C); IR (KBr) 3053, 3021, 2917, 2856, 2359, 1882, 1784, 1505, 1292, 811 cm^−1^; ^1^H NMR (400 MHz, CDCl_3_) *δ* 2.22 (s, 6H), 6.95–7.04 (m, 9H), 7.24–7.27 (m, 1H), 7.50 (td, 1H), 7.73–7.79 (m, 2H), 8.32–8.35 (m, 1H), 8.50–8.51 (m, 1H), 8.53–8.55 (m, 1H); ^13^C NMR (500 MHz, CDCl_3_) *δ* 20.8, 119.7, 112.1, 112.2, 122.7, 124.4, 129.8, 133.0, 136.1, 136.9, 144.7, 146.3, 147.5, 149.32, 149.39, 151.5, 151.8, 162.8; MS (EI) *m*/*z* 434 (M^+^); HRMS (EI) calcd for C_27_H_22_N_4_S, 434.1565; found, 434.1549.

### 2-(5-Methylpyridin-2-yl)-4-(pyridin-2-yl)-*N*,*N*-di-*p*-tolylthiazol-5-amine (4c)

#### General procedure A

4c (0.077 g, 59%) as a yellow solid (mp: 116–117 °C); IR (KBr) 1686, 1605, 1383, 1314, 1287, 1002, 810 cm^−1^; ^1^H NMR (400 MHz, CDCl_3_) *δ* 2.23 (s, 6H) 2.36 (s, 3H) 6.95–6.95 (m, 1H) 6.98 (m, 6H) 7.01 (t, 1H) 7.04–7.07 (m, 1H) 7.51–7.53 (m, 1H) 7.57 (dd, 1H) 7.78 (d, *J* = 7.79 Hz, 1H) 8.26–8.27 (m, 1H) 8.33 (d, *J* = 1.83 Hz, 1H) 8.58 (s, 1H); ^13^C NMR (400 MHz, CDCl_3_) *δ* 18.6, 20.8, 119.0, 119.2, 121.2, 122.1, 122.7, 129.7, 132.8, 134.4, 136.0, 137.3, 144.6, 149.1, 149.51, 149.6, 151.9, 161.8, 163.3; MS (EI) *m*/*z* 434 (M^+^); HRMS (EI) calcd for C_28_H_24_N_4_S, 448.1722; found, 448.1716.

### 2-Phenyl-4-(pyridin-2-yl)-*N*,*N*-di-*p*-tolylthiazol-5-amine (4d)

#### General procedure A

4d (0.171 g, 59%) as a yellow solid (mp: 135–136 °C); IR (KBr) 3420, 1507, 1475, 1426, 1314, 1290, 814, 761, 686 cm^−1^; ^1^H NMR (400 MHz, CDCl_3_) *δ* 2.29 (s, 6H), 7.03–7.05 (m, 4H), 7.07–7.09 (m, 4H), 7.42–7.45 (m, 2H), 7.47–7.48 (m, 2H), 8.02–8.03 (d, *J* = 3.7 Hz, 2H), 8.22 (d, *J* = 6.4, 2H), 9.09–9.10 (d, *J* = 3.7 Hz, 1H); ^13^C NMR (400 MHz, CDCl_3_) *δ* 20.8, 102.3, 118.1, 122.1, 122.4, 122.8, 126.6, 127.7, 128.8, 129.9, 130.2, 133.4, 144.4, 150.4, 156.3, 162.8; MS (EI) *m*/*z* 433 (M^+^); HRMS (EI) calcd for C_28_H_23_N_3_S, 433.1613; found, 433.1612.

### *N*,*N*,2-Triphenyl-4-(pyridin-2-yl)thiazol-5-amine (4e)

#### General procedure A

4e (0.064 g, 32%) as a yellow ocher solid (mp: 174–175 °C); IR (KBr) 2359, 2334, 1734, 1716, 1698, 1684, 1653, 1583, 1541, 1519, 1490, 1422, 1352, 1312, 1284, 1233, 846, 729, 757, 698 cm^−1^; ^1^H NMR (400 MHz, CDCl_3_) *δ* 6.89 (t, 2H), 6.94–6.98 (m, 1H), 7.06–7.14 (m, 9H), 7.32–7.34 (m, 2H), 7.45 (td, 1H), 7.74 (d, *J* = 7.8, 1H), 7.89–7.92 (m, 2H), 8.42–8.44 (m, 1H) cm^−1^; ^13^C NMR (400 MHz, CDCl_3_) *δ* 121.9, 122.1, 122.6, 123.0, 126.4, 128.7, 129.0, 120.0, 133.7, 135.9, 143.6, 146.6, 147.4, 149.4, 151.9, 163.0; MS (EI) *m*/*z* 433 (M^+^); HRMS (EI) calcd for C_26_H_19_N_3_S, 405.1300; found, 405.1281.

### 4-Phenyl-2-(pyridin-2-yl)-*N*,*N*-di-*p*-tolylthiazol-5-amine (4f)

#### General procedure A

4f 4(0.0219 g, 90%) as a greenish yellow solid (mp: 192–193 °C); IR (KBr) 3421, 2921, 1586, 1569, 1506, 1470, 1424, 12 990, 1002, 811, 508 cm^−1^; ^1^H NMR (400 MHz, CDCl_3_) *δ* 2.24 (s, 6H), 6.96–7.04 (m, 8H), 7.18–7.22 (tt, 1H), 7.24–7.25 (m, 1H), 7.26–7.28, (dd, 1H), 7.31–7.34 (td, 1H), 7.81–7.85 (td, 1H), 7.93–7.96 (dd, 2H), 8.29 (d, *J* = 7.79 Hz, 1H) 8.56 (d, *J* = 4.58 Hz, 1H); ^13^C NMR (400 MHz, CDCl_3_) *δ* 20.7, 119.6, 121.75, 124.4, 127.4, 127.9, 128.2, 129.8, 132.7, 133.5, 137.5, 143.8, 144.54, 148.4, 148.8, 151.2; MS (EI) *m*/*z* 433 (M^+^); HRMS (EI) calcd for C_28_H_23_N_3_S, 433.1613; found, 433.1621.

### 2-(4-Methoxyphenyl)-4-(pyridin-2-yl)-*N*-(*p*-tolyl)thiazol-5-amine (6a)

#### General procedure B

6a (0.66 g, 60%) as a pale yellow solid (mp: 136 °C); IR (ATR) 2996, 1589, 1565, 1547, 1513, 1395, 1320, 1294, 1240, 1167, 1146, 1025, 971, 827, 782, 703, 493 cm^−1^; ^1^H NMR (400 MHz, CDCl_3_) *δ* 2.33 (s, 3H), 3.84 (s, 3H) 6.93 (d, *J* = 8.53 Hz, 2H), 7.07–7.24 (m, 5H), 7.74 (t, 1H), 7.82–7.85 (m, 2H), 8.23 (s, 1H), 8.52 (d, *J* = 4.49 Hz, 1H), 11.5 (s, 1H); ^13^C NMR (400 MHz, CDCl_3_) *δ* 20.8, 55.4, 114.3, 117.8, 119.7, 120.8, 127.0, 130.0, 136.8, 139.9, 146.8, 147.1, 160.3; MS (EI) *m*/*z* 373(M^+^); HRMS (EI) calcd for C_22_H_19_N_3_OS, 373.1249; found, 373.1245.

### *N*-(4-(*Tert*-butyl)phenyl)-2-(4-methoxyphenyl)-4-(pyridin-2-yl)thiazol-5-amine (6b)

#### General procedure B

6b (0.42 g, 49%) as a pale-yellow solid (mp: 120 °C); IR (ATR) 2956, 1587, 1563, 1535, 1471, 1387, 1245, 1170, 1032, 825, 782, 705, 541, 513 cm^−1^; ^1^H NMR (400 MHz, CDCl_3_) *δ* 1.35 (s, 9H), 3.83 (s, 3H) 6.93 (d, *J* = 8.98 Hz, 2H), 7.04–7.07 (m, 1H), 7.24–7.28 (m, 2H), 7.40 (d, *J* = 8.08 Hz, 2H), 7.72 (t, 1H), 7.84 (d, *J* = 8.53 Hz, 2H), 8.12–8.28 (m, 1H), 8.51 (d, *J* = 4.94 Hz, 1H), 11.6 (s, 1H); ^13^C NMR (400 MHz, CDCl_3_) *δ* 31.5, 34.0, 55.4, 111.9, 112.3, 114.3, 117.2, 117.3, 119.6, 120.7, 125.7, 126.3, 127.0, 130.3, 130.7, 136.6, 139.7, 145.0, 145.4, 147.1, 149.0, 155.8, 160.3; MS (EI) *m*/*z* 415(M^+^); HRMS (EI) calcd for C_25_H_25_N_3_OS, 415.1718; found, 415.1705.

### Complex [Ni(4a)Cl] (7a)

#### General procedure C

5a (0.0355 g, 86%) as a green solid (mp: 264–265 °C); IR (KBr) 3274, 1597, 1545, 1507, 1463, 1374, 1078, 786 cm^−1^; ESI-MS calcd for C_15_H_14_Cl_2_N_4_NiS, 374.9981; found 375.8088.

### Complex [Ni(4b)Cl] (7b)

#### General procedure C

7b (0.05 g, 85%) (mp: 193–194 °C); IR (KBr) 3421, 2921, 2852, 1597, 1506, 1455, 1262, 811, 778, 676 cm^−1^; ESI-MS calcd for C_27_H_22_Cl_2_N_4_NiS, 562.0607; found 562.0301.

### Complex [Ni(4c)Cl] (7c)

#### General procedure C

7c (0.024 g, 88%) (mp: 306–307 °C); IR (KBr) 3370, 1600, 1506, 1455, 1267, 813 cm^−1^; ESI-MS calcd for C_28_H_24_Cl_2_N_4_NiS, 541.0764; found 542.2366.

### Complex [Ni(4d)Cl] (7d)

#### General procedure C

7d (0.066 g, 83%) (mp: 292–293 °C); IR (KBr) 3383, 2921, 1707, 1603, 1473, 1361, 1238, 813, 682, 566 cm^−1^; ESI-MS calcd for C_56_H_46_ClN_6_NiS_2_, 959.2267; found 959.0425.

### Complex [Ni(4e)Cl] (7e)

#### General procedure C

7e (0.035 g, 97%) (mp: 177–178 °C); IR (KBr) 3384, 1602, 1489, 1360, 1237, 753, 694 cm^−1^; ESI-MS calcd for C_52_H_38_Cl_2_N_6_NiS_2_, 903.1641; found 903.0425.

### Complex [Ni(4f)Cl] (7f)

#### General procedure C

7f (0.05 g, 97%) (mp: 292–293 °C); IR (KBr) 3370, 1600, 1506, 1455, 1267, 813 cm^−1^; ESI-MS calcd for C_56_H_46_ClN_6_NiS_2_, 959.2267; found 961.1050.

### Complex [Zn(4f)_2_Cl_2_] (8a)

#### General procedure D

8a (0.079 g, 93%) as a red solid (mp: 230–233 °C); IR (ATR) 1600, 1505, 1474, 1454, 1357, 1259, 811, 780, 753, 698, 501 cm^−1^; ^1^H NMR (500 MHz, CDCl_3_) *δ* 2.25 (s, 6H), 6.90 (d, *J* = 8.53 Hz, 4H), 7.00 (d, *J* = 8.08 Hz, 4H), 7.26–7.26 (m, 3H) 7.63–7.66 (m, 1H), 7.69–7.71 (m, 1H), 7.74 (dd, 2H), 8.04 (td, 1H), 8.74 (d, *J* = 4.94 Hz, 1H); ^13^C NMR (400 MHz, CDCl_3_) *δ* 20.9, 25.6, 68.2, 121.5, 122.8, 126.9, 128.5, 128.8, 129.6, 129.8, 130.2, 134.8, 141.1, 143.3, 145.3, 145.6, 147.1, 148.8, 154.7; ESI-MS calcd for C_56_H_46_ClN_6_S_2_Zn^+^, 965.2205; found 965.2498.

### Complex [Zn(4f)_2_Br_2_] (8b)

#### General procedure D

8b (0.072 g, 73%) as a yellow solid (mp: >250 °C); IR (ATR) 3027, 2969, 1738, 1602, 1539, 1506, 1476, 1455, 1364, 1228, 1216, 1011, 807, 776, 700, 556, 510, 408 cm^−1^; ^1^H NMR (500 MHz, CDCl_3_) *δ* 2.25 (s, 6H), 6.90 (d, *J* = 8.53 Hz, 4H), 7.00 (d, *J* = 8.08 Hz, 4H), 7.26–7.26 (m, 3H) 7.63–7.66 (m, 1H), 7.69–7.71 (m, 1H), 7.74 (dd, 2H), 8.04 (td, 1H), 8.74 (d, *J* = 4.94 Hz, 1H); ^13^C NMR (400 MHz, CDCl_3_) *δ* 20.8, 121.5, 122.8, 126.9, 128.5, 128.8, 129.6, 129.8, 130.2, 134.8, 141.1, 143.3, 145.3, 145.6, 147.1, 148.8, 154.7; ESI-MS calcd for C_56_H_46_BrN_6_S_2_Zn^+^, 1012.4300; found 1012.2454.

### Complex [Zn(4f)_2_I_2_] (8c)

#### General procedure D

8c (0.076 g, 67%) as a yellow solid (mp: >250 °C); IR (ATR) 3026, 2969, 1738, 1597, 1506, 1472, 1449, 1369, 1228, 1216, 1159, 1012, 810, 771, 700, 573, 513, 411 cm^−1^; ^1^H NMR (500 MHz, CDCl_3_) *δ* 2.25 (s, 6H), 6.90 (d, *J* = 8.53 Hz, 4H), 7.00 (d, *J* = 8.08 Hz, 4H), 7.26–7.26 (m, 3H) 7.63–7.66 (m, 1H), 7.69–7.71 (m, 1H), 7.74 (dd, 2H), 8.04 (td, 1H), 8.74 (d, *J* = 4.94 Hz, 1H); ^13^C NMR (400 MHz, CDCl_3_) *δ* 20.8, 121.5, 122.8, 126.9, 128.5, 128.8, 129.6, 129.8, 130.2, 134.8, 141.1, 143.3, 145.3, 145.6, 147.1, 148.8, 154.7; ESI-MS calcd for C_56_H_46_IN_6_S_2_Zn^+^, 1057.1562; found 1057.2929.

## Author contributions

Khurnia Krisna Puji Pamungkas synthesized compounds, studied their photophysical properties and wrote the manuscript, Toshifumi Maruyama performed and determined X-ray structure, and Toshiaki Murai, supervised the project and wrote the manuscript.

## Conflicts of interest

There are no conflicts to declare.

## Supplementary Material

RA-012-D2RA01694J-s001

RA-012-D2RA01694J-s002

## References

[cit1] Ji Ram V. , Sethi A., Nath M. and Pratap R., in The Chemistry of Heterocycles, Elsevier, 2019, pp. 149–478

[cit2] Sharma P. C., Bansal K. K., Sharma A., Sharma D., Deep A. (2020). Eur. J. Med. Chem..

[cit3] Liu H., Xu L., Hui H., Vivian R., Callebaut C., Murray B. P., Hong A., Lee M. S., Tsai L. K., Chau J. K., Stray K. M., Cannizzaro C., Choi Y. C., Rhodes G. R., Desai M. C. (2014). Bioorg. Med. Chem. Lett..

[cit4] Shreykar M. R., Sekar N. (2017). Dyes Pigm..

[cit5] Lugovik K. I., Popova A. V., Eltyshev A. K., Benassi E., Belskaya N. P. (2017). Eur. J. Org. Chem..

[cit6] Gayathri P., Kanagajothi K., Nag P., Anand N., Reddy V. S., Moon D., Anthony S. P., Madhu V. (2021). CrystEngComm.

[cit7] Suntsova P. O., Eltyshev A. K., Pospelova T. A., Slepukhin P. A., Benassi E., Belskaya N. P. (2019). Dyes Pigm..

[cit8] Sun H., Sun W.-H., Jiang Y., Wei J.-H., Zhao Y., Zhang R., Ni Z.-H. (2020). Dyes Pigm..

[cit9] Puji Pamungkas K. K., Maruyama T., Murai T. (2021). Org. Biomol. Chem..

[cit10] Potopnyk M. A., Lytvyn R., Danyliv Y., Ceborska M., Bezvikonnyi O., Volyniuk D., Gražulevičius J. V. (2018). J. Org. Chem..

[cit11] Lakowicz J. R. (2001). Anal. Biochem..

[cit12] Jeong Y., Kook Y.-M., Lee K., Koh W.-G. (2018). Biosens. Bioelectron..

[cit13] Abd El Aleem Ali Ali El-Remaily M., El-Dabea T., Alsawat M., Mahmoud M. H. H., Abdulaziz Alfi A., El-Metwaly N., Abu-Dief A. M. (2021). ACS Omega.

[cit14] El-Remaily M. A. E. A. A. A., Soliman A. M. M., Khalifa M. E., El-Metwaly N. M., Alsoliemy A., El-Dabea T., Abu-Dief A. M. (2022). Appl. Organomet. Chem..

[cit15] Zou X., Shi P., Feng A., Mei M., Li Y. (2021). Transition Met. Chem..

[cit16] Zou X.-Z., Feng A.-S., Liao Y.-Z., Xu X.-Y., Wen H.-Y., You A., Mei M., Li Y. (2020). Inorg. Chem. Commun..

[cit17] Menzel R., Kupfer S., Mede R., Weiß D., Görls H., González L., Beckert R. (2012). Eur. J. Org. Chem..

[cit18] Huffman S. E., Yawson G. K., Fisher S. S., Bothwell P. J., Platt D. C., Jones M. A., Hamaker C. G., Webb M. I. (2020). Metallomics.

[cit19] Thangadurai T. D., Ihm S.-K. (2005). Synth. React. Inorg., Met.-Org., Nano-Met. Chem..

[cit20] Liu Y., Sun X., Wang Y., Wu Z. (2014). Synth. Met..

[cit21] Hallett A. J., Placet E., Prieux R., McCafferty D., Platts J. A., Lloyd D., Isaacs M., Hayes A. J., Coles S. J., Pitak M. B., Marchant S., Marriott S. N., Allemann R. K., Dervisi A., Fallis I. A. (2018). Dalton Trans..

[cit22] Leopold H., Tronnier A., Wagenblast G., Münster I., Strassner T. (2016). Organometallics.

[cit23] Chikineva T. Y., Koshelev D. S., Medved'ko A. V., Vashchenko A. A., Lepnev L. S., Goloveshkin A. S., Utochnikova V. V. (2021). Russ. J. Inorg. Chem..

[cit24] Dannenbauer N., Matthes P. R., Scheller T. P., Nitsch J., Zottnick S. H., Gernert M. S., Steffen A., Lambert C., Müller-Buschbaum K. (2016). Inorg. Chem..

[cit25] Luconi L., Lyubov D. M., Rossin A., Glukhova T. A., Cherkasov A. V., Tuci G., Fukin G. K., Trifonov A. A., Giambastiani G. (2014). Organometallics.

[cit26] Büldt L. A., Larsen C. B., Wenger O. S. (2017). Chem.–Eur. J..

[cit27] Núñez C., Bastida R., MacÍas A., Valencia L., Ribas J., Capelo J. L., Lodeiro C. (2010). Dalton Trans..

[cit28] Meundaeng N., Rujiwatra A., Prior T. J. (2016). Transition Met. Chem..

[cit29] Rossin A., Di Credico B., Giambastiani G., Gonsalvi L., Peruzzini M., Reginato G. (2011). Eur. J. Inorg. Chem..

[cit30] Helal A., Kim H. S. (2010). Tetrahedron.

[cit31] Ergun E., Ergun Ü., İleri Ö., Küçükmüzevir M. F. (2018). Spectrochim. Acta, Part A.

[cit32] Mikata Y., Takekoshi A., Kaneda M., Konno H., Yasuda K., Aoyama M., Tamotsu S. (2017). Dalton Trans..

[cit33] Mehta P. K., Oh E. T., Park H. J., Lee K. H. (2017). Spectrochim. Acta, Part B.

[cit34] Yu F., Guo X., Tian X., Jia L. (2017). J. Fluoresc..

[cit35] Yun J. Y., Kim A., Hwang S. M., Yun D., Lee H., Kim K.-T., Kim C. (2019). Bull. Chem. Soc. Jpn..

[cit36] Aydin D., Karuk Elmas S. N., Savran T., Arslan F. N., Sadi G., Yilmaz I. (2021). J. Photochem. Photobiol., A.

[cit37] Watanabe Y., Sungnoi W., Sartorio A. O., Zeller M., Wei A. (2020). Mater. Chem. Front..

[cit38] Yamaguchi K., Murai T., Hasegawa S., Miwa Y., Kutsumizu S., Maruyama T., Sasamori T., Tokitoh N. (2015). J. Org. Chem..

[cit39] Yamaguchi K., Murai T., Guo J. D., Sasamori T., Tokitoh N. (2016). ChemistryOpen.

[cit40] Yamaguchi K., Murai T., Tsuchiya Y., Miwa Y., Kutsumizu S., Sasamori T., Tokitoh N. (2017). RSC Adv..

[cit41] Murai T., Furukawa H., Yamaguchi K. (2018). Heterocycles.

[cit42] Murai T., Yoshihara M., Yamaguchi K., Minoura M. (2019). Asian J. Org. Chem..

[cit43] Murai T., Nakatsu Y., Tsuchiya Y., Yamaguchi K., Maruyama T., Miwa Y., Kutsumizu S. (2020). Heterocycles.

[cit44] Tsuchiya Y., Yamaguchi K., Miwa Y., Kutsumizu S., Minoura M., Murai T. (2020). Bull. Chem. Soc. Jpn..

