# Supplementary material for: 5-N-Arylaminothiazoles with pyridyl groups and their first-row transition metal complexes: synthesis, photophysical properties, and Zn sensing
Source: RSC Adv. 2022 May 16;12(23):14698–706. doi: 10.1039/d2ra01694j (PMC9109715; doi:10.1039/d2ra01694j)

Electronic Supplementary Material (ESI) for RSC Advances.
This journal is © The Royal Society of Chemistry 2022

Supporting Information

5-*N*-Arylaminothiazoles with pyridyl groups and their first-row transition metal complexes: synthesis, photophysical properties, and Zn sensing

Khurnia Krisna Puji Pamungkas, Toshifumi Maruyama and Toshiaki Murai*

Department of Chemistry and Biomolecular Science, Faculty of Engineering, Gifu University, Yanagido, Gifu 501-1193, Japan.

Email: mtoshi@gifu-u.ac.jp

Contents

General Procedure for the Preparation of Thiazolines. 3

Photophysical properties of 5-*N*-arylaminothiazoles 4

Fig. S1 Absorption (left) and emission properties of **4a**. 4

Table S1 Spectroscopic data of **4a**. 4

Fig. S2 Absorption (left) and emission (right) spectra of **4b**. 5

Table S2 Spectroscopic data of **4b**. 5

Fig. S3 Absorption (left) and emission (right) spectra of **4c**. 6

Table S3 Spectroscopic data of **4c**. 6

Fig. S4 Absorption (left) and emission (right) spectra of **4d**. 7

Table S4 Spectroscopic data of **4d**. 7

Fig. S*5* Absorption (left) and emission (right) spectra of **4e**. 8

Table S5 Spectroscopic data of **4e**. 8

Fig. S6 Absorption (left) and emission (right) spectra of **4f**. 9

Table S6 Spectroscopic data of **4f**. 9

Spectroscopic data of zinc-thiazole 6a in different solvent polarities 10

Table S7 Spectroscopic data of zinc complex **8a** in different solvent polarities. 10

X-ray crystallography data of thiazole and nickel-thiazole complex. 11

Table S8. X-ray crystallography data of thiazole **4c**. 11

Table S9 X-ray crystallography data of nickel-thiazole complex **7c**. 12

^{1}H NMR charts for 5-aminothiazoles, dipyrromethene type ligands, and zinc-thiazole complexes. 16

^{13}C NMR charts for 5-aminothiazoles and zinc-thiazole complexes. 27

General Procedure for the Preparation of Thiazolines.

To a solution of thioamides (1 equiv) in THF was added slowly a 1.25 M solution of *n*-buthyllithium in *n*-hexane (2 equiv) at 0 ˚C, and the mixture was stirred for 10 minutes in this temperature. To this was added thioformamides (1 equiv) at 0 ˚C, and the mixture was stirred for 10 minutes at this temperature. To this was added iodine (2-3 equiv) at 0 ˚C, and the mixture was stirred for 2 h at 0 ˚C. The resulting mixture was poured into a saturated aqueous solution of $Na_2S_2O_3$ and extracted with Et_2O. The organic layer was dried over $MgSO_4$ and concentrated in vacuo. The residue was purified by column chromatography (SiO_2) to give the corresponding thiazolines.

N,N-dimethyl-2,4-di(pyridin-2-yl)thiazol-5-amine (3a)
Directly converted to thiazole **4a.**

2,4-Di(pyridin-2-yl)-N,N-di-p-tolyl-4,5-dihydrothiazol-5-amine (3b)
8b (0.304 g, 46%) as a ocher yellow solid (mp: 70-72 ºC); IR (KBr) 3049, 1585, 1567, 1505, 1464, 1434, 1422, 1294, 1002, 810, 781, 736 cm^{-1} ; ^1H NMR (400 MHz, $CDCl_3$) δ 2.27 (s, 6H, Me) 6.12 (d, *J* = 4.3 Hz, 1H) 6.74 (d, *J* = 4.8 Hz, 1H) 6.97-6.99 (m, 4H) 7.04-7.05 (m, 4H) 7.21 (d, *J* = 7.7 Hz, 1H) 7.23-7.24 (m, 1H), 7.35-7.38 (m, 1H) 7.60-7.63 (m, 1H), 7.73 (t, 1H), 7.95 (d, *J* = 7.7 Hz, 1H), 8.64-8.68 (dd, 2H); ^{13}C NMR (500 MHz, $CDCl_3$) δ 20.8, 78.7, 84.2, 121.5, 122.5, 123.6, 125.5, 129.4, 129.8, 133.0, 136.5, 136.7, 143.8, 149.3, 149.8, 151.6, 159.1, 171.8; MS (EI) *m/z* 436 (M^+); HRMS (EI) calcd for $C_{27}H_{24}N_4S$, 436.1722; found, 436.1690.

2-(5-Methylpyridin-2-yl)-4-(pyridin-2-yl)-N,N-di-p-tolyl-4,5-dihydrothiazol-5-amine (3c)
3c (0.154 g, 95%) as a ocher yellow solid (mp: 51-52 ºC); IR (KBr) 3419 3082, 2916, 1609, 1588, 1517, 1470, 1319, 1001, 808, 504 cm^{-1} ; ^1H NMR (400 MHz, $CDCl_3$) δ 2.25 (s, 6H), 2.37 (s, 3H), 6.09 (d, *J* = 4.1 Hz, 1H), 6.70 (d, *J* = 4.1 Hz, 1H), 6.91 (d, *J* = 5.5 Hz, 1H), 6.97-6.98 (m, 2H), 7.01-7.03 (m, 5H), 7.17-7.21 (m, 2H), 7.50 (d, *J* = 5.5 Hz, 1H), 7.57-7.61 (m, 1H), 7.83 (d, *J* = 7.8 Hz, 1H), 8.46-8.47 (m, 1H), 8.62 (d, *J* = 4.1 Hz, 1H) ; ^{13}C (400 MHz, $CDCl_3$) δ 20.72, 117.9, 119.4, 199.9, 121.3, 122.9, 124.7, 129.8, 130.1, 134.7, 137.1, 137.3, 137.5, 141.1, 148.9, 149.2, 149.5, 149.9, 152.4; MS (EI) *m/z* 450 (M^+); HRMS (EI) calcd for $C_{28}H_{26}N_4S$, 450.1878; found, 450.1720.

2-Phenyl-4-(pyridin-2-yl)-N,N-di-p-tolyl-4,5-dihydrothiazol-5-amine (3d)
3d (0.295 g, 67%) as a yellow solid (mp: 43-44 ºC); IR (KBr) 3025, 2919, 1586, 1508, 1432, 1231, 1039, 949, 808, 765, 690, 570 cm^{-1} ; ^1H NMR (400 MHz, $CDCl_3$) δ 2.26 (s, 6H), 6.10 (d, *J* = 3.2 Hz, 1H), 6.76 (d, *J* = 3.2 Hz, 1H), 6.95-6.98 (m, 4H), 7.03-7.05 (m, 4H), 7.17-7.22 (m, 2H), 3.75-3.79 (m, 2H), 7.43-7.46 (m, 1H), 7.57-7.62 (td, 1H), 7.78-7.81 (m, 2H), 8.62-8.63 (m, 1H); ^{13}C (400 MHz, $CDCl_3$) δ 20.8, 79.7, 83.6, 117.9, 121.4, 122.7, 123.3, 128.4, 128.5, 129.9, 131.3, 133.2, 133.8, 136.7, 143.5, 149.6, 159.1 ; MS (EI) *m/z* 435(M^+); HRMS (EI) calcd for $C_{28}H_{25}N_3S$, 435.1769; found, 435.1770.

***N,N,*2-triphenyl-4-(pyridin-2-yl)-4,5-dihydrothiazol-5-amine (3e)**
3e (0.181 g, 447%) as a yellow solid (mp: 98-99 ºC); IR (KBr) 3063, 2360, 2332, 1653, 1586, 1489, 1434, 1230, 1094, 1041, 956, 751, 692, 624, 591, 555, 531 cm^{-1}; ^1H NMR (400 MHz, $CDCl_3$) δ 6.16 (d, *J* = 2.9 Hz, 1H), 6.83 (d, *J* = 3.9 Hz, 1H), 7.04 (t, 2H), 7.08-7.10 (m, 4H), 7.14-7.26 (m, 6H), 7.35 (t, 2H), 7.40-7.44 (m, 1H), 7.56 (dd, 1H), 7.79 (d, *J* = 7.3 Hz, 2H), 8.63 (d, *J* = 4.9 Hz, 1H); ^{13}C (400 MHz, $CDCl_3$) δ 79.1, 83.7, 121.1, 122.6, 123.4, 123.6, 128.2, 128.3, 129.2, 131.2, 133.6, 145.7, 158.9, 169.6; MS (EI) *m/z* 407(M^+); HRMS (EI) calcd for $C_{26}H_{21}N_3S$, 407.1456; found, 407.1433.

4-Phenyl-2-(pyridin-2-yl)-N,N-di-p-tolyl-4,5-dihydrothiazol-5-amine (8f)
8f (1.482 g, 68%) as a yellow solid (mp: 50-51 ºC); IR (KBr) 3421, 2920, 1604, 1508, 1240, 1033, 964, 788, 730, 574 cm^{-1}; ^1H NMR (400 MHz, $CDCl_3$) δ 2.26 (s, 6H), 5.99 (d, *J* = 4.6 Hz, 1H), 6.29 (d, *J* = 4.6 Hz, 1H), 6.87 (d, *J* = 4.1 Hz, 1H), 6.95-6.97 (m, 3H), 7.05 (d, *J* = 8.2 Hz, 4H), 7.27-7.28 (m, 1H), 7.34 (d, *J* = 4.1 Hz, 4H), 3.37-3.39 (m, 1H), 7.72-7.76 (td, 1H), 8.08 (d, *J* = 7.8 Hz, 1H), 8.68 (d, *J* = 5.5 Hz, 1H); ^{13}C (400 MHz, $CDCl_3$) δ 20.28, 80.77, 123.22, 123.66, 124.58, 126.56, 128.13, 128.63, 128.98, 129.42, 130.05, 133.39, 136.76, 143.71, 146.17, 149.46, 151.07; MS (EI) *m/z* 435(M^+); HRMS (EI) calcd for $C_{28}H_{25}N_4S$, 435.1769; found, 435.1799.

Photophysical properties of 5-*N*-arylaminothiazoles

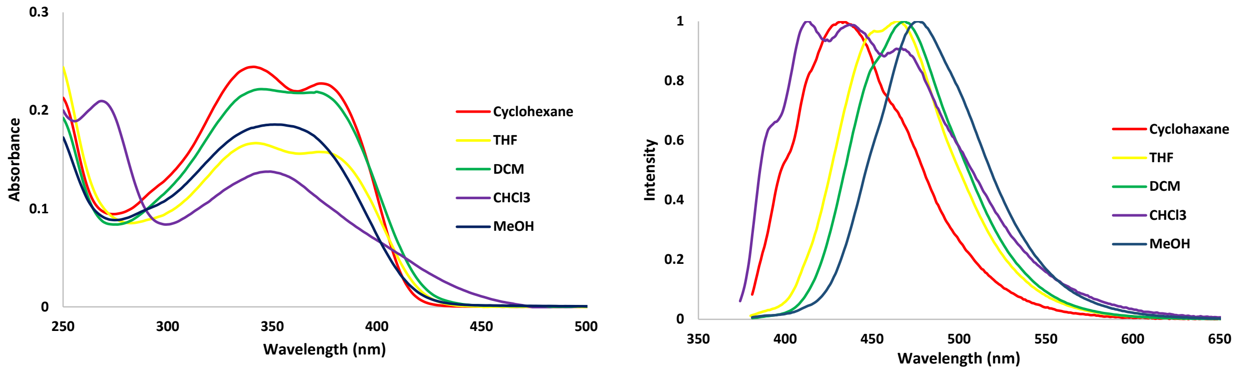

Fig. S1 Absorption (left) and emission properties of **4a**.

Table S1 Spectroscopic data of **4a**.

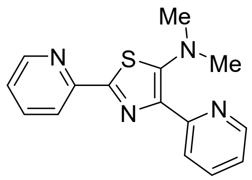

4a

Thiazole	Solv.	UV-Vis[a]		Flourescence[b]			Stoke shift
		λ_{abs}(nm)	logε	λ_{ex}(nm)	λ_{em} (nm)	Φ_F[b,c]	(cm^{-1}) [nm]
**4a**	Solid	434		435	475	0.03	1988 [41]
	Cyclohexane	341<br>**373**	4.36	288<br>342 (s)<br>**366**	**432**<br>465 (s)	0.03	3661 [59]
	THF	342<br>**372**	4.19	342 (s)<br>**365**	452 (s)<br>**464**	0.05	5330 [92]
	DCM	345<br>371	4.34	366	468	0.05	5586 [97]
	$CHCl_3$	268<br>**349**	4.14	292<br>**359**	413<br>439<br>**467**	0.04	7240 [118]
	MeOH	351	4.26	366	476	0.04	7481 [125]

[solute] = 10^{-5}M   [a]Measured on V-770   [b]Measured on FP-8500   [c]Excited at λ_{ex}

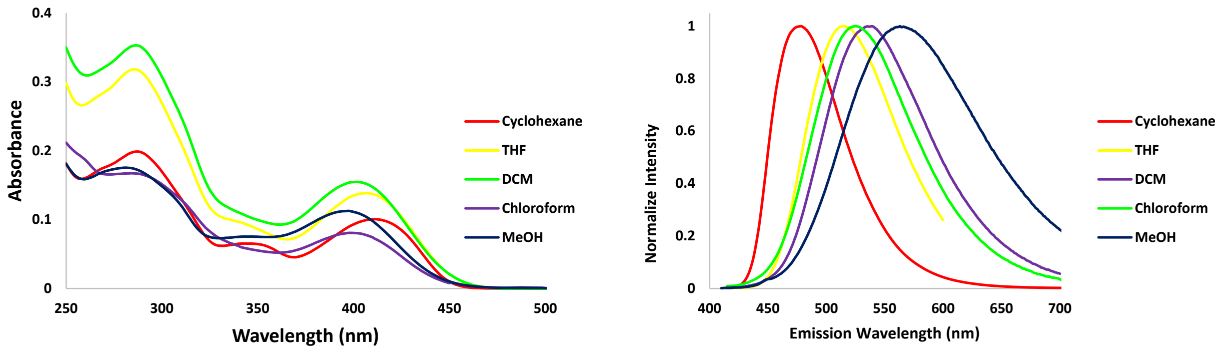

Fig. S2 Absorption (left) and emission (right) spectra of **4b**.

Table S2 Spectroscopic data of **4b**.

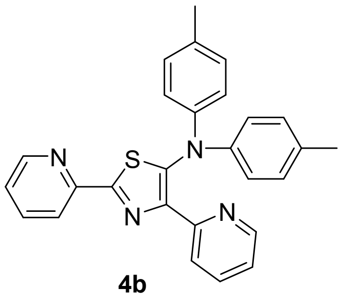

4b

Thiazole	Solv.	UV-Vis[a]		Flourescence[b]			Stoke shift
		λ_{abs}(nm)	logε	λ_{ex}(nm)	λ_{em} (nm)	Φ_F[b,c]	(cm^{-1}) [nm]
4b	Solid	442		439	495	0.21	2411 [53]
	Cyclohexane	411	4.30	410	478	0.41	3410 [67]
	THF	407	4.14	406	515	0.21	5152 [108]
	DCM	402	4.19	398	539	0.16	6323 [137]
	$CHCl_3$	398	3.90	397	525	0.13	6078 [127]
	MeOH	397	4.26	395	563	0.15	7427 [166]

[solute] = 10^{-5}M [a]Measured on V-770 [b]Measured on FP-8500 [c]Excited at λ_{ex}

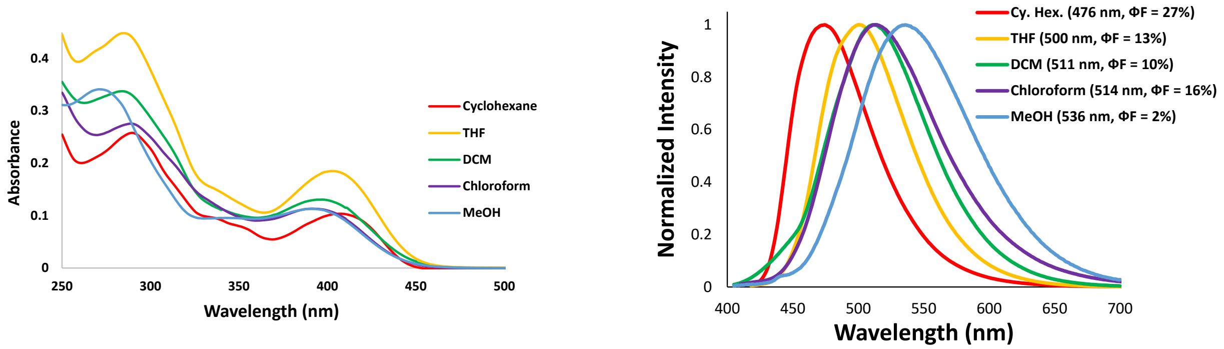

Fig. S3 Absorption (left) and emission (right) spectra of **4c**.

Table S3 Spectroscopic data of **4c**.

Thiazole	Solv.	UV-Vis[a]		Flourescence[b]			Stoke shift
		λ_{abs}(nm)	logε	λ_{ex}(nm)	λ_{em} (nm)	Φ_F[b,c]	(cm^{-1}) [nm]
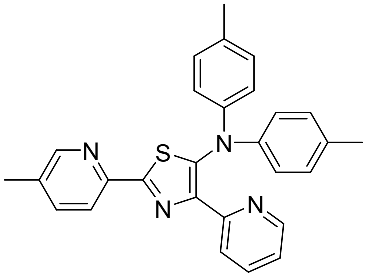 **4c**	Solid	444	-	437	524	0.06	3438 [80]
	Cyclohexane	240<br>407	4.01	283<br>407	474	0.27	3473 [67]
	THF	285<br>403	4.26	284<br>398	500	0.13	4813 [97]
	DCM	284<br>397	4.11	280<br>389	511	0.10	5619 [113]
	$CHCl_3$	289<br>391	4.05	252<br>285<br>395	514	0.16	6120 [123]
	MeOH	271<br>391	4.05	279<br>389	536	0.02	6918 [145]

[solute] = 10^{-5}M [a]Measured on V-770 [b]Measured on FP-8500 [c]Excited at λ_{ex}

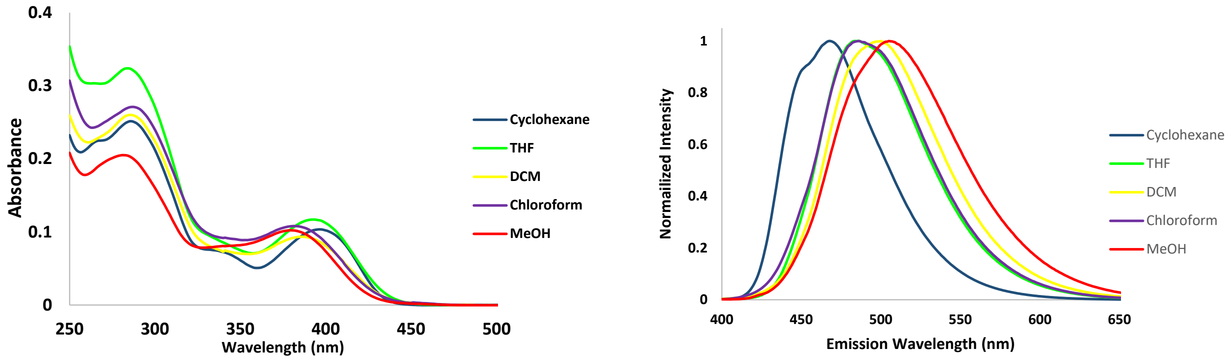

Fig. S4 Absorption (left) and emission (right) spectra of **4d**.

Table S4 Spectroscopic data of **4d**.

Thiazole	Solv.	UV-Visa		Flourescenceb			Stoke shift (cm-1) [nm]
		λ_{abs}(nm)	logε	λ_{ex}(nm)	λ_{em} (nm)^{b,c}	Φ_F^{b,c}	
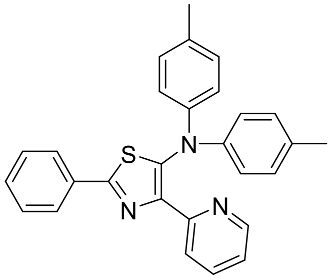 **4d**	Solid	444		279<br>369<br>437	475	0.21	1469 [31]
	Cyclohexane	286<br>397	4.01	282<br>393	468	0.29	3821 [71]
	THF	284<br>393	4.06	281<br>390	486	0.17	4869 [93]
	DCM	286<br>387	3.97	282<br>380	500	0.15	5839 [93]
	$CHCl_3$	287<br>381	4.03	284<br>377	486	0.20	5670 [113]
	MeOH	281<br>379	4.00	278<br>375	505	0.12	6583 [126]

[solute] = 10^{-5}M [a]Measured on V-770 [b]Measured on FP-8500 [c]Excited at λ_{ex}

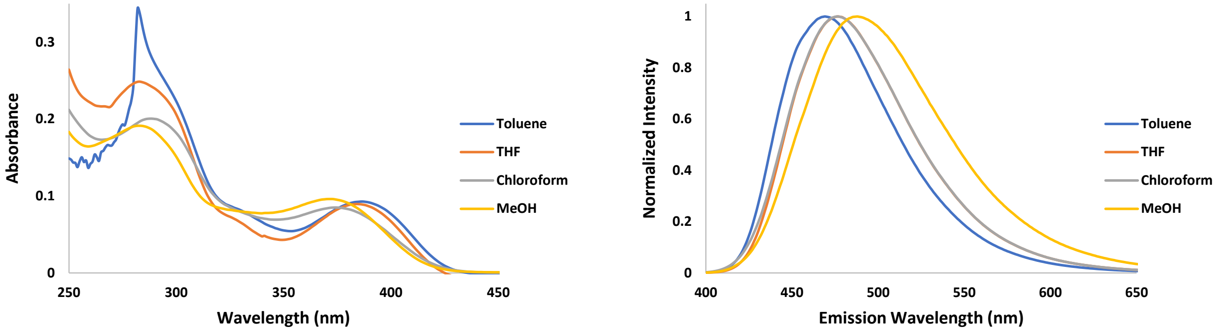

Fig. S5 Absorption (left) and emission (right) spectra of **4e**.

Table S5 Spectroscopic data of **4e**.

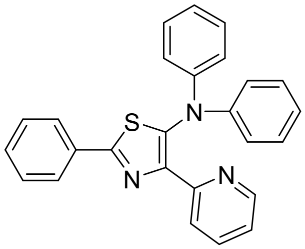

4e

Thiazole	Solv.	UV-Vis[a]		Flourescence[b]			Stoke shift
		λ_{abs}(nm)	logε	λ_{ex}(nm)	λ_{em} (nm)	Φ_F[b,c]	(cm^{-1}) [nm]
**4e**	Solid	427		326<br>363<br>428	469	0.15	2097 [42]
	Toluene	282<br>386	3.96	297<br>387	469	0.26	4584 [83]
	THF	283<br>**384**	3.95	283<br>**383**	477	0.26	5077 [93]
	$CHCl_3$	288<br>**374**	3.92	376	476	0.31	5729 [102]
	MeOH	283<br>372	3.98	282<br>371	488	0.8	6389 [116]

[solute] = 10^{-5}M [a]Measured on V-770 [b]Measured on FP-8500 [c]Excited at λ_{ex}

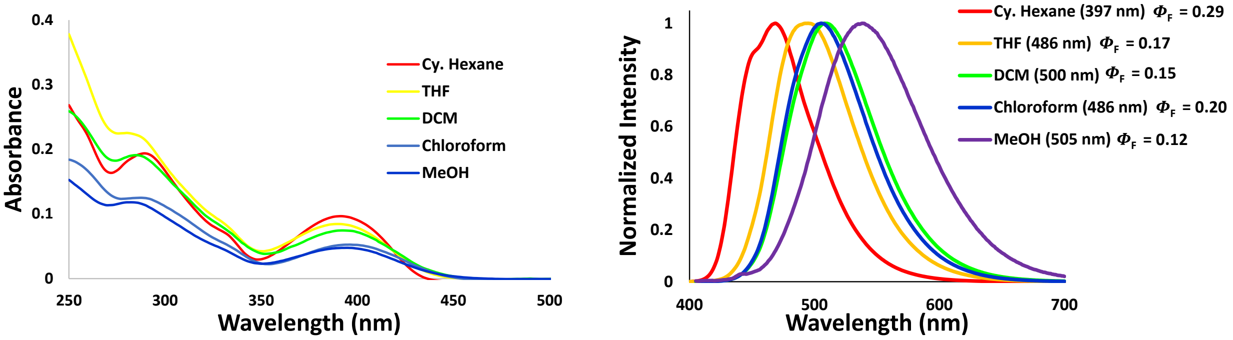

Fig. S6 Absorption (left) and emission (right) spectra of **4f**.

Table S6 Spectroscopic data of **4f**.

Thiazole	Solv.	UV-Vis[a] λ_{abs}(nm)	UV-Vis[a] logε	Flourescence[b] λ_{ex}(nm)	Flourescence[b] λ_{em} (nm)	Flourescence[b] Φ_F[b,c]	Stoke shift (cm^{-1}) [nm]
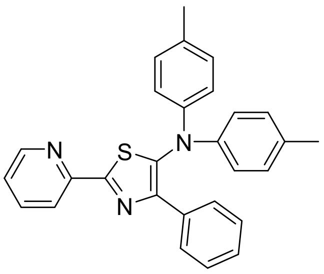 **4f**	Solid	404		275<br>365<br>431	475	0.52	
	Cyclohexane	289<br>**391**		282<br>**387**	469	0.44	
	THF	280<br>**390**	3.92	281<br>**385**	495	0.46	5439
	DCM	285<br>392	3.87	281<br>388	508	0.39	5825
	$CHCl_3$	287<br>**396**	3.72	285<br>**392**	505	0.42	5450
	MeOH	282<br>395	3.67	258<br>281<br>389	538	0.12	6729

[solute] = 10^{-5}M [a]Measured on V-770 [b]Measured on FP-8500 [c]Excited at λ_{ex}

Spectroscopic data of zinc-thiazole 8a in different solvent polarities

Table S7 Spectroscopic data of zinc complex **8a** in different solvent polarities.

Complex	Solvent	**Uv**	λ_{ex}	λ_{em}	υ_{ss}(nm)	Φ_F
**8a**	$CHCl_3$	274<br>463	463	590	127	0.36
	DCM	271<br>459	458	608	149	0.31
	THF	282<br>390	384	497	107	0.46
	MeOH	282<br>391	388	538	147	0.66

Conc. 10^{-5} M

X-ray crystallography data of thiazole and nickel-thiazole complex.

Table S8. X-ray crystallography data of thiazole **4c**.

Chemical formula	$C_{28}H_{24}N_4S$
M_r	448.57
Crystal system, space group	Triclinic, $P\bar{1}$
Temperature (K)	193
a, *b*, *c* (Å)	9.407 (3), 11.689 (4), 12.393 (4)
α, β, γ (°)	115.971 (4), 102.307 (2), 91.501 (2)
V (Å^3)	1185.8 (7)
Z	2
Radiation type	Mo $K\alpha$
μ (mm^{-1})	0.16
Crystal size (mm)	0.43 × 0.37 × 0.34
Diffractometer	Rigaku Mercury CCD (2x2 bin mode)
Absorption correction	Numerical
T_{min}, T_{max}	0.872, 0.921
No. of measured, independent and observed [$I > 2\sigma(I)$] reflections	10688, 5374, 3854
R_{int}	0.027
$(\sin \theta/\lambda)_{max}$ (Å^{-1})	0.650
$R[F^2 > 2\sigma(F^2)]$, $wR(F^2)$, S	0.047, 0.135, 0.97
No. of reflections	5374
No. of parameters	301
H-atom treatment	H-atom parameters constrained
$\Delta□_{max}$, $\Delta□_{min}$ (e Å^{-3})	0.25, -0.32

Table S9 X-ray crystallography data of nickel-thiazole complex **7c**.

Chemical formula	$C_{28}H_{24}Cl_2N_4NiS$
M_r	578.18
Crystal system, space group	Monoclinic, $P2_1/n$
Temperature (K)	293
a, *b*, *c* (Å)	15.9050 (11), 8.6642 (4), 20.5670 (9)
β (°)	107.528 (6)
V (Å^3)	2702.6 (3)
Z	4
Radiation type	Mo $K\alpha$
μ (mm^{-1})	1.02
Crystal size (mm)	0.17 × 0.11 × 0.11
Diffractometer	Rigaku Mercury CCD (2x2 bin mode)
Absorption correction	Numerical
T_{min}, T_{max}	0.846, 0.896
No. of measured, independent and observed [$I > 2\sigma(I)$] reflections	19039, 4754, 3743
R_{int}	0.102
$(\sin \theta/\lambda)_{max}$ (Å^{-1})	0.650
$R[F^2 > 2\sigma(F^2)]$, $wR(F^2)$, S	0.068, 0.148, 1.087
No. of reflections	4754
No. of parameters	328
H-atom treatment	H-atom parameters constrained
Δ□$_{max}$, Δ□$_{min}$ (e Å^{-3})	0.84, -0.74

ESI-Mass analysis spectra

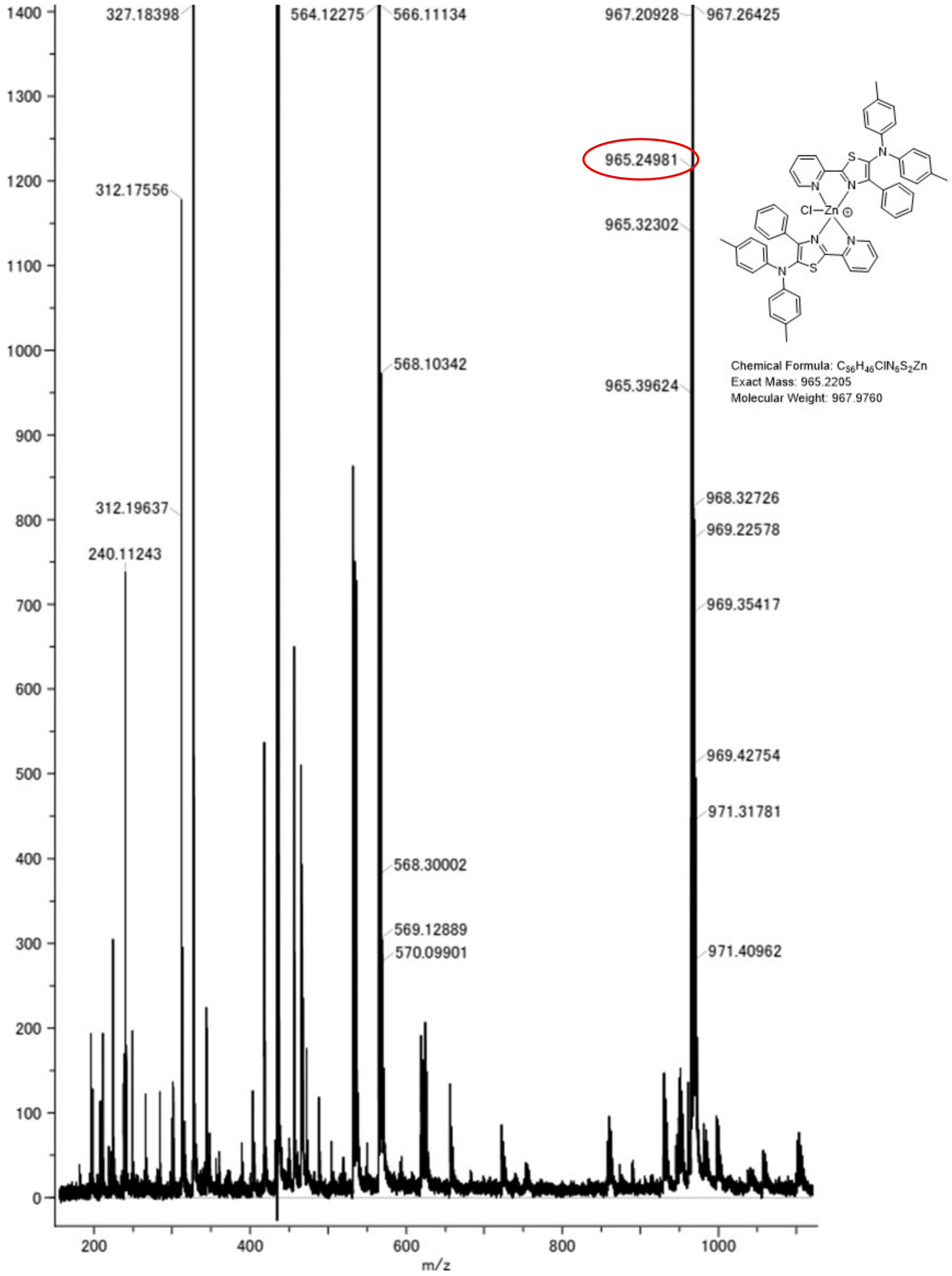

Fig. S7 ESI-Mass analysis spectra of **8a**.

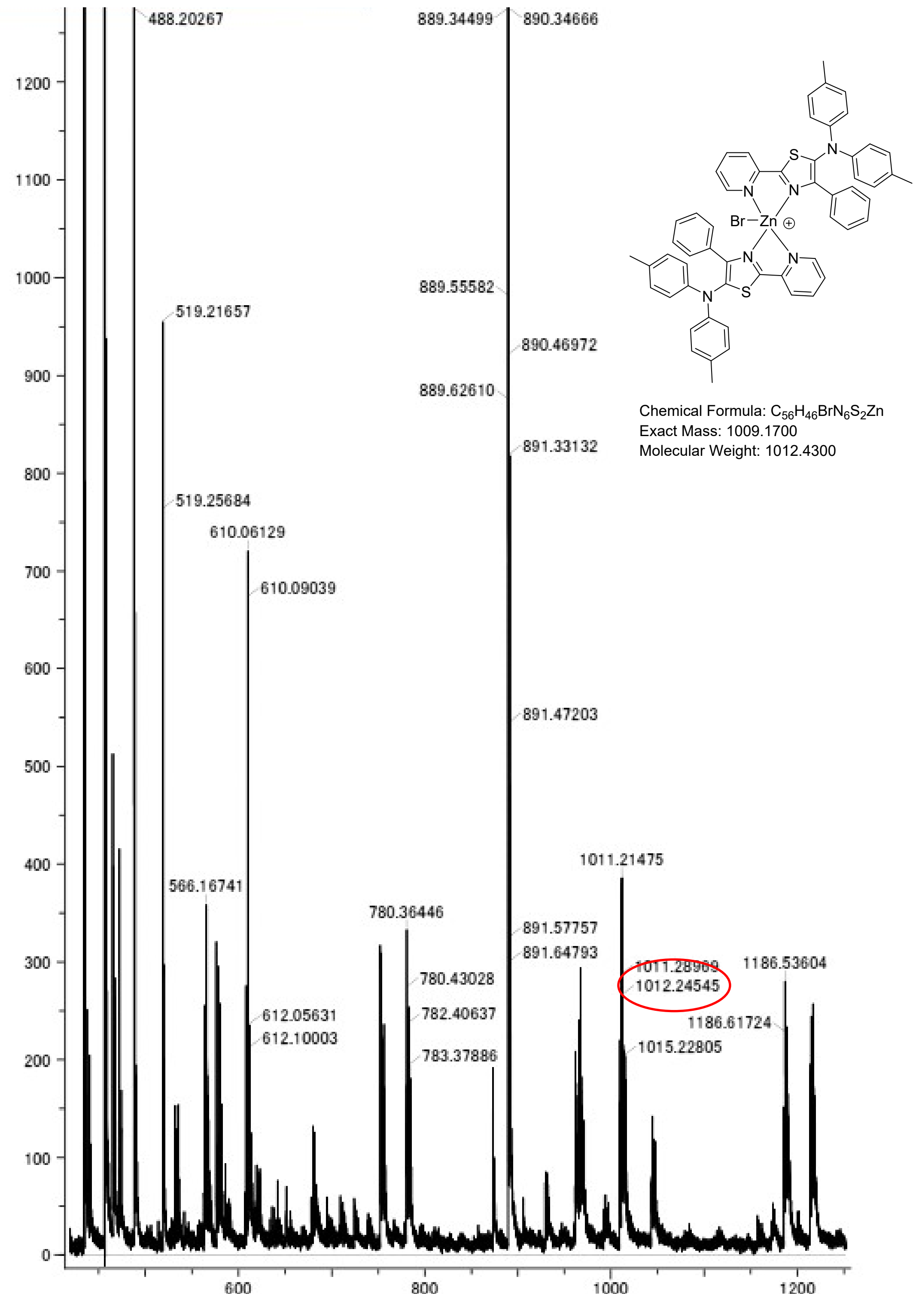

Fig. S8. ESI-Mass analysis of **8b**.

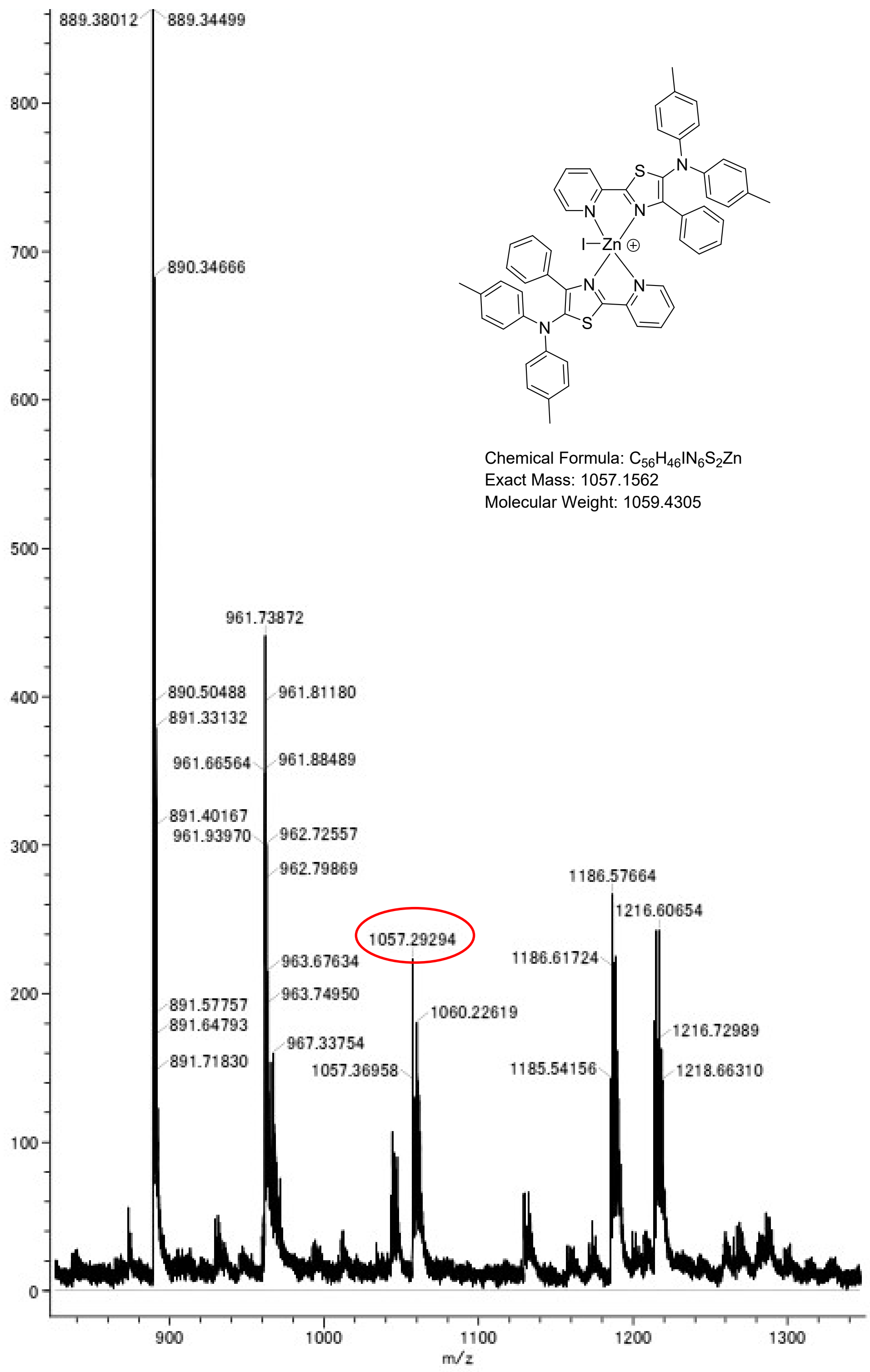

Fig. S9. ESI-Mass analysis of **8c**.

^{1}H NMR charts for 5-*N*-arylaminothiazoles, dipyrromethene type ligands, and zinc-thiazole complexes.

5-aminothiazole **4a**

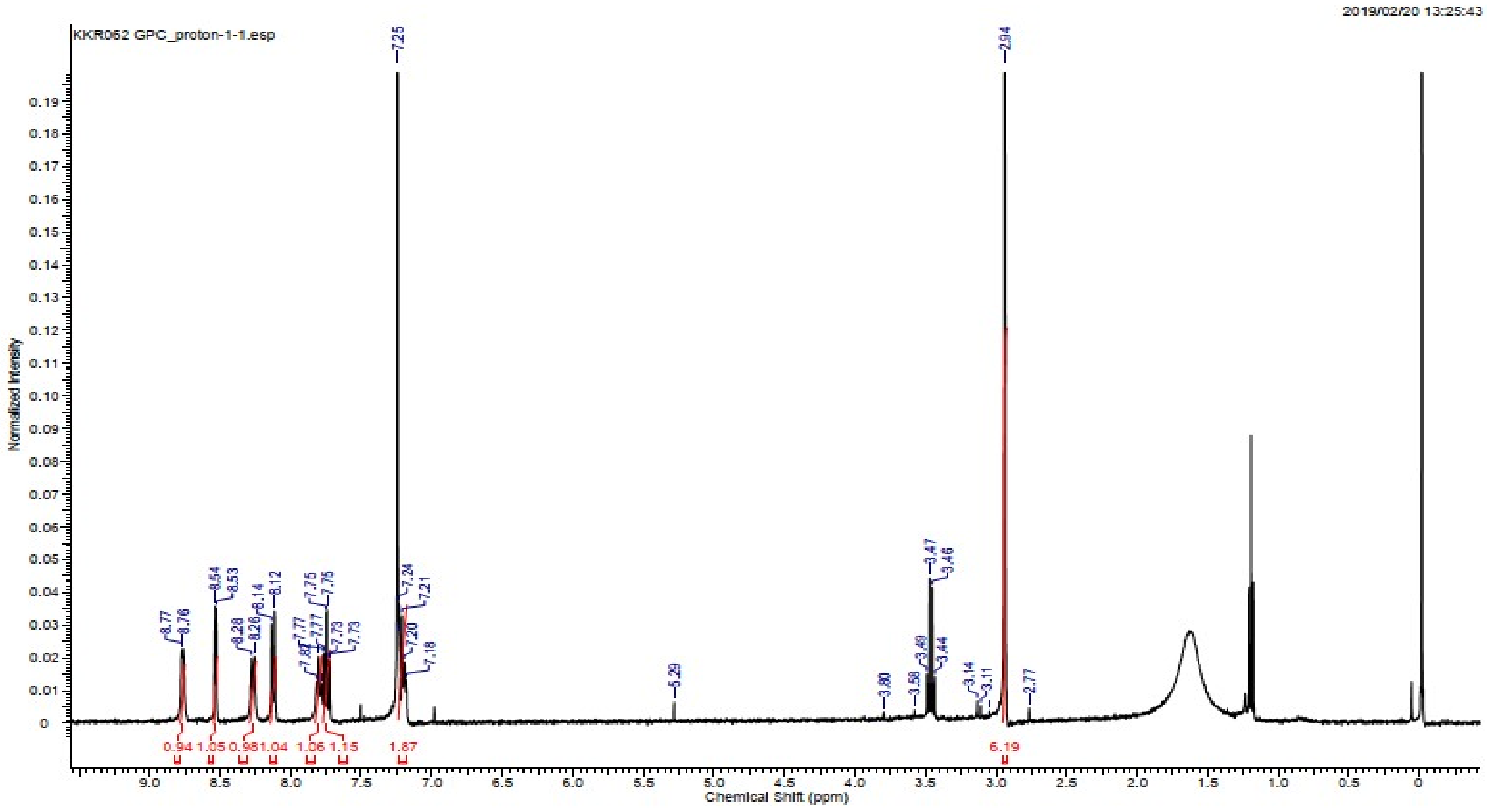

5-aminothiazole **4b**

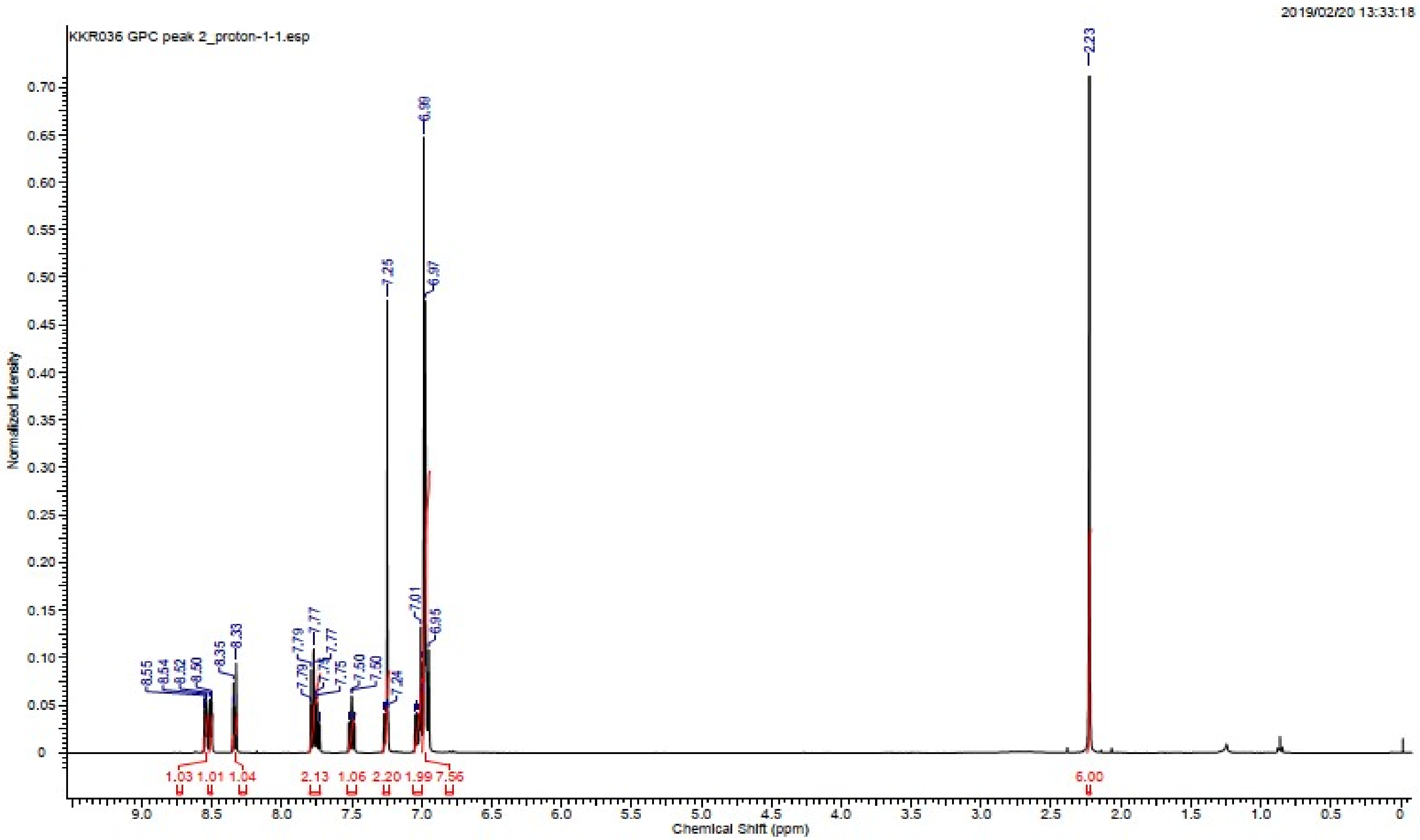

5-aminothiazole **4c**

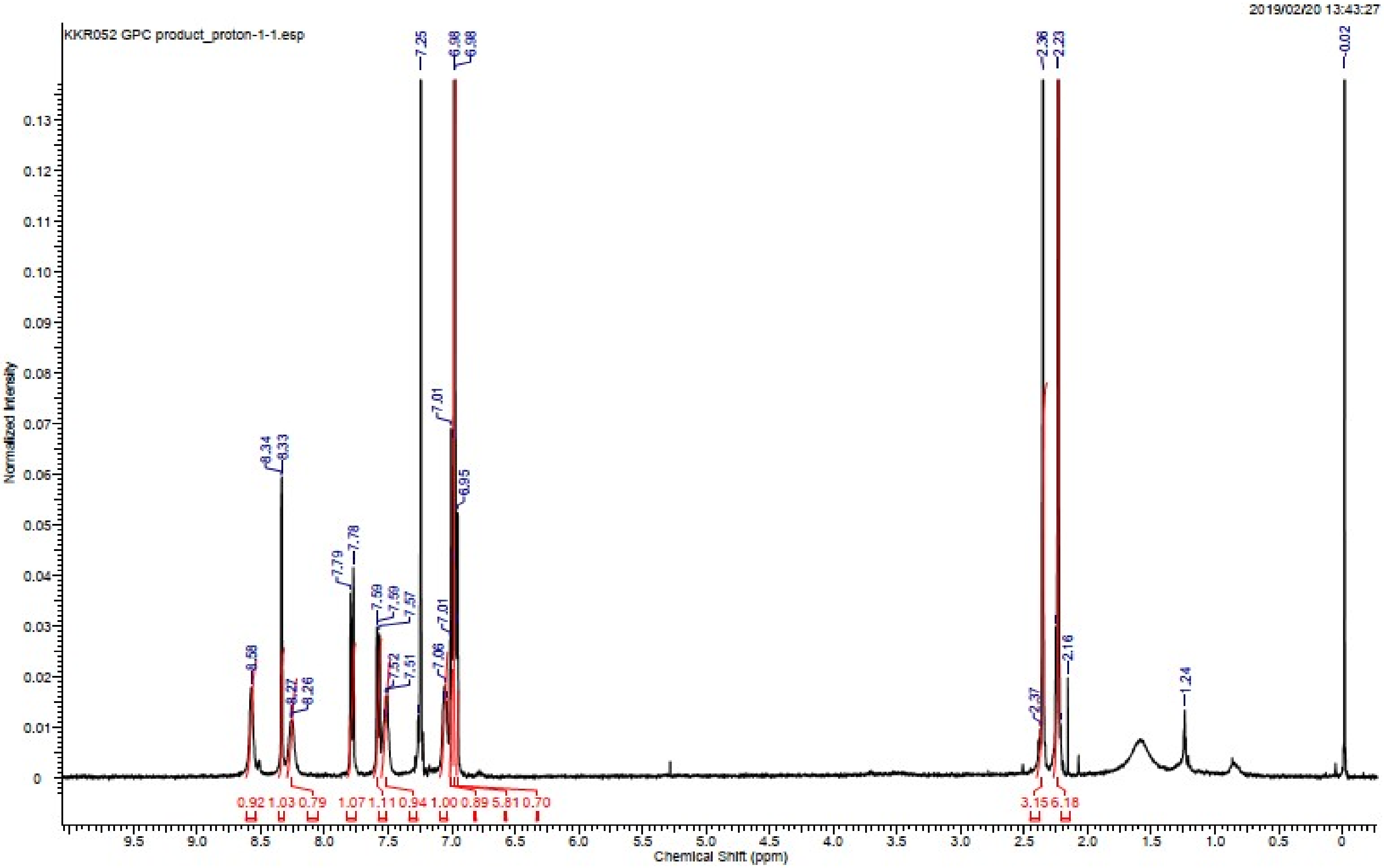

5-aminothiazole **4d**

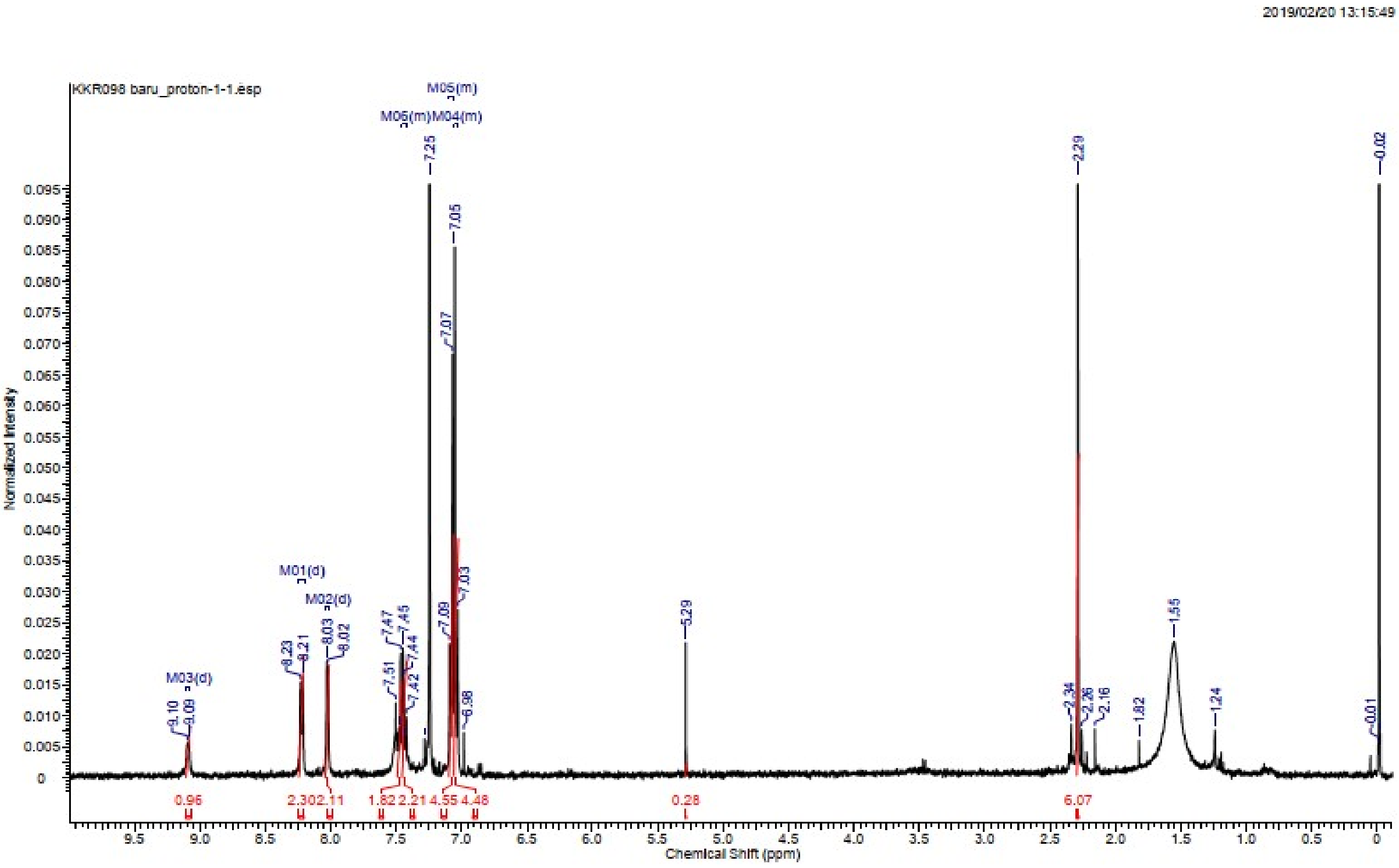

5-aminothiazole **4e**

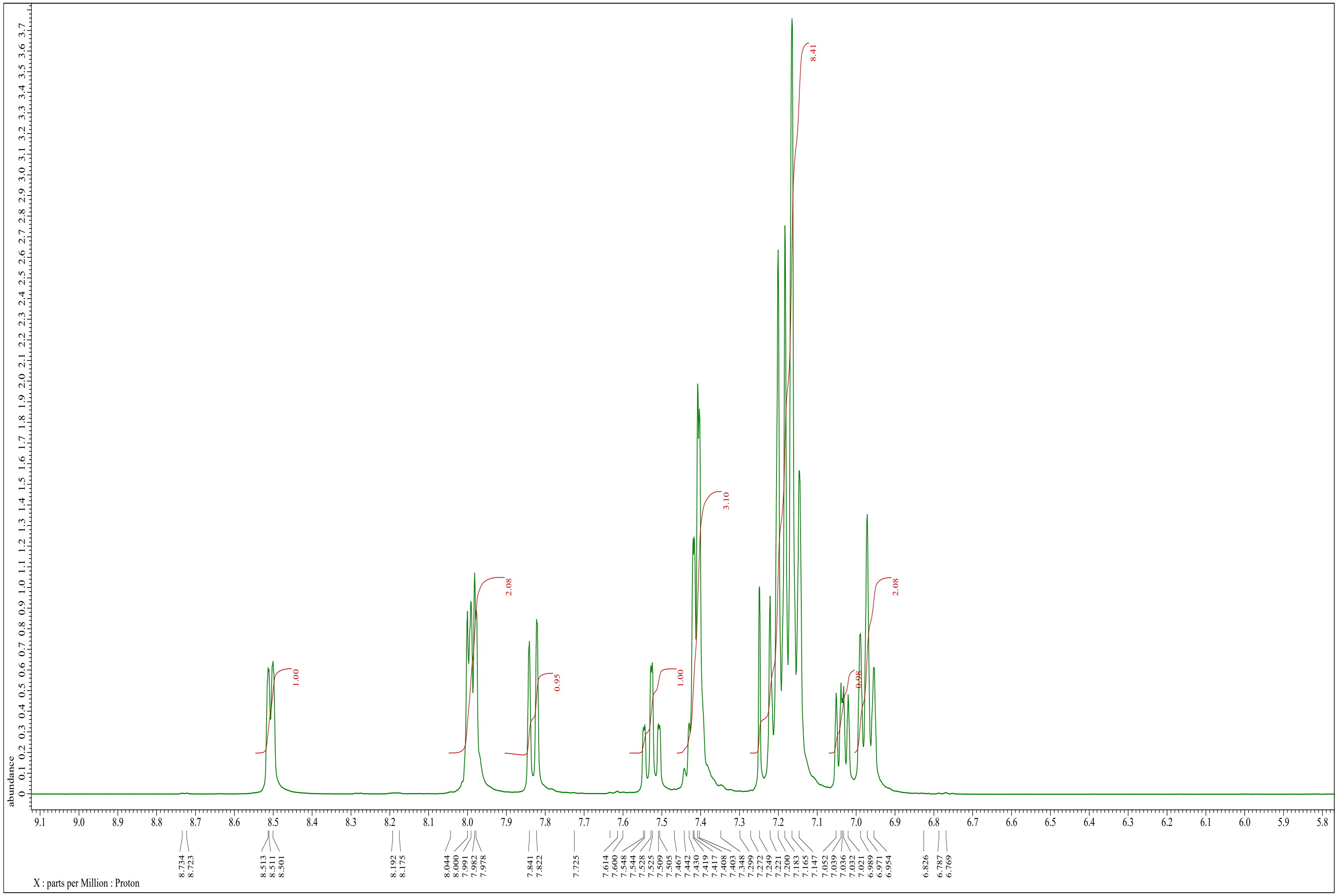

5-aminothiazole **4f**

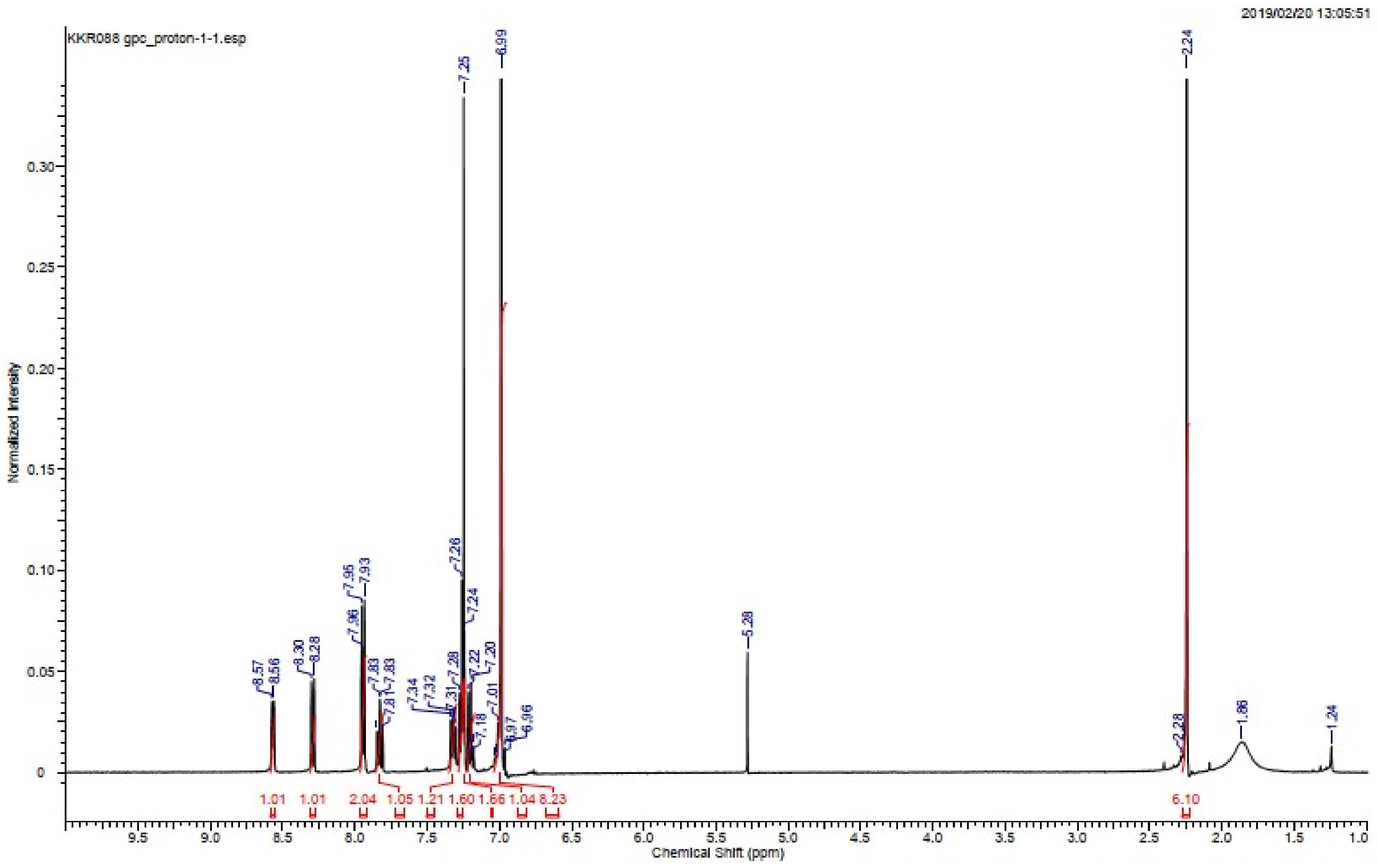

Dipyrromethene type ligand **6a**

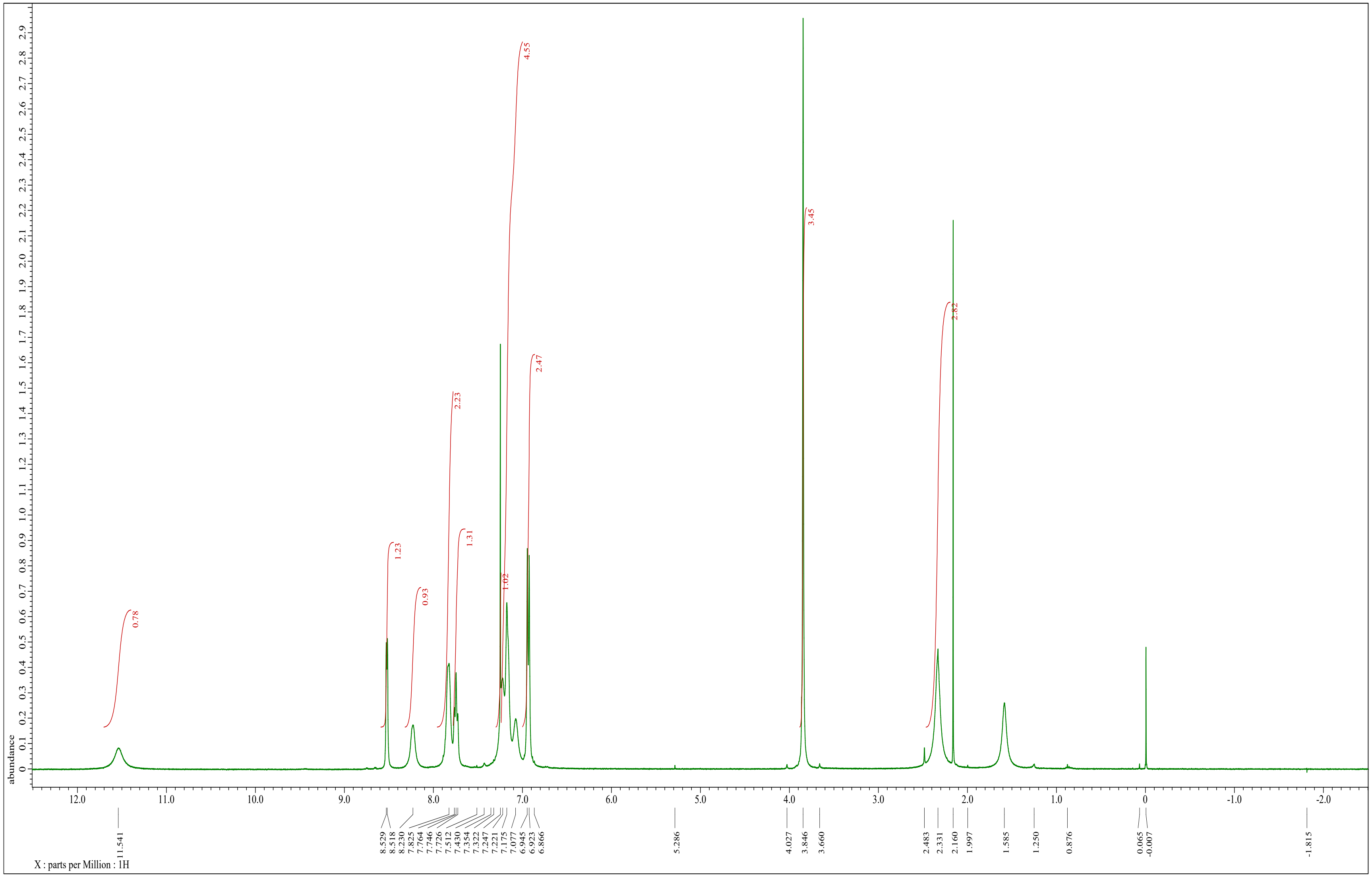

Dipyrromethene type ligand **6b**

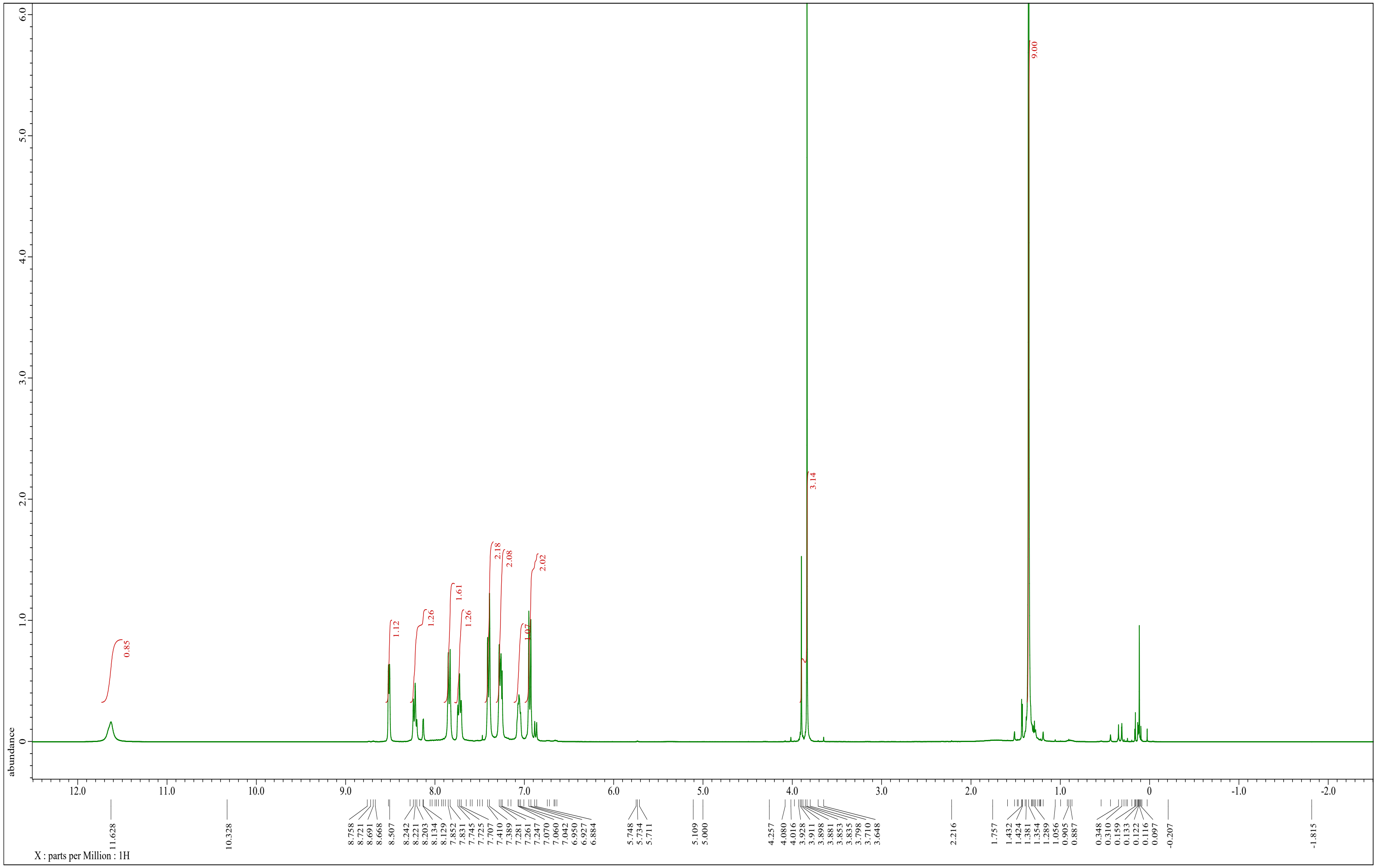

Zinc-thiazole complex **8a**

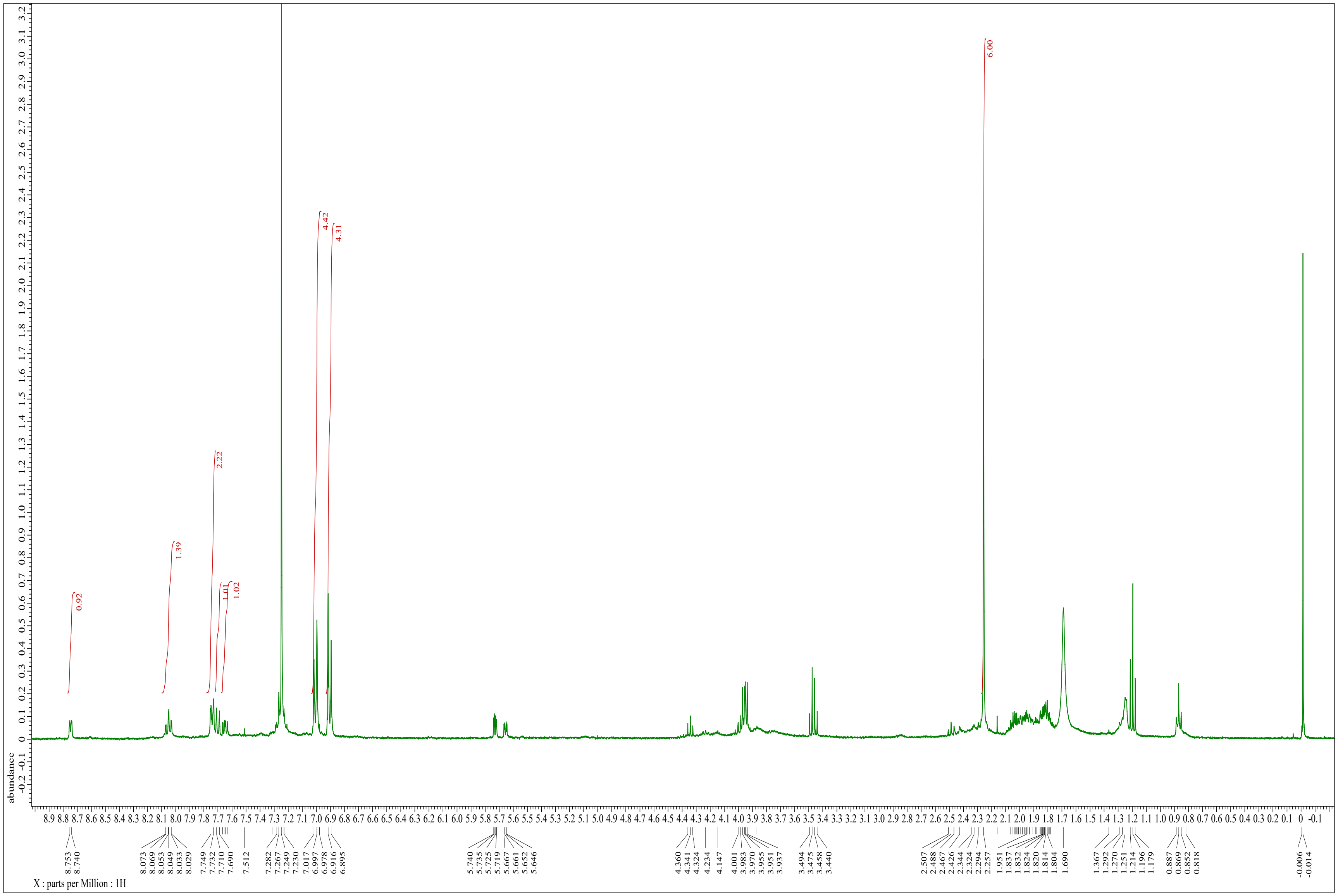

Zinc-thiazole complex **8b**

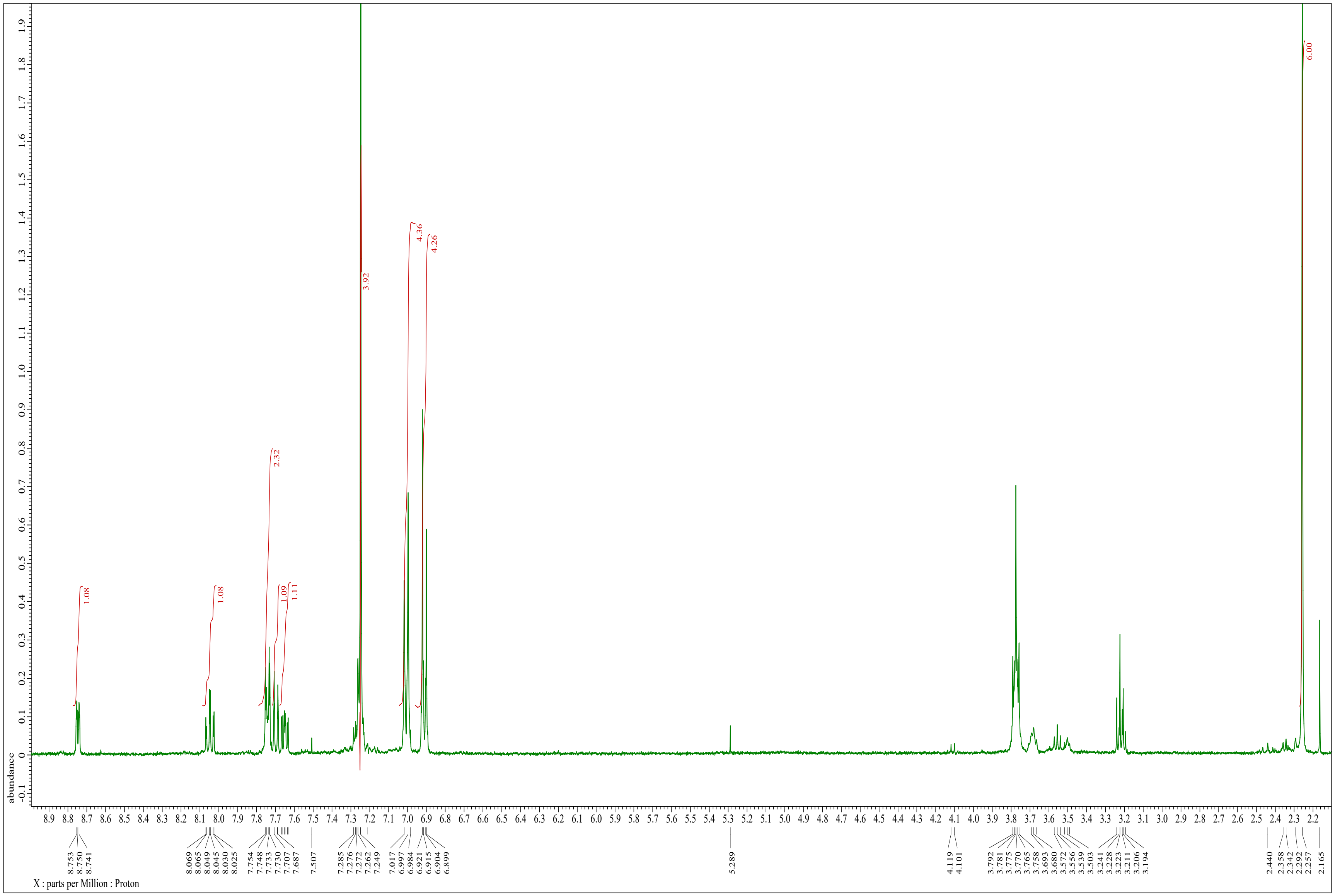

Zinc-thiazole complex **8c**

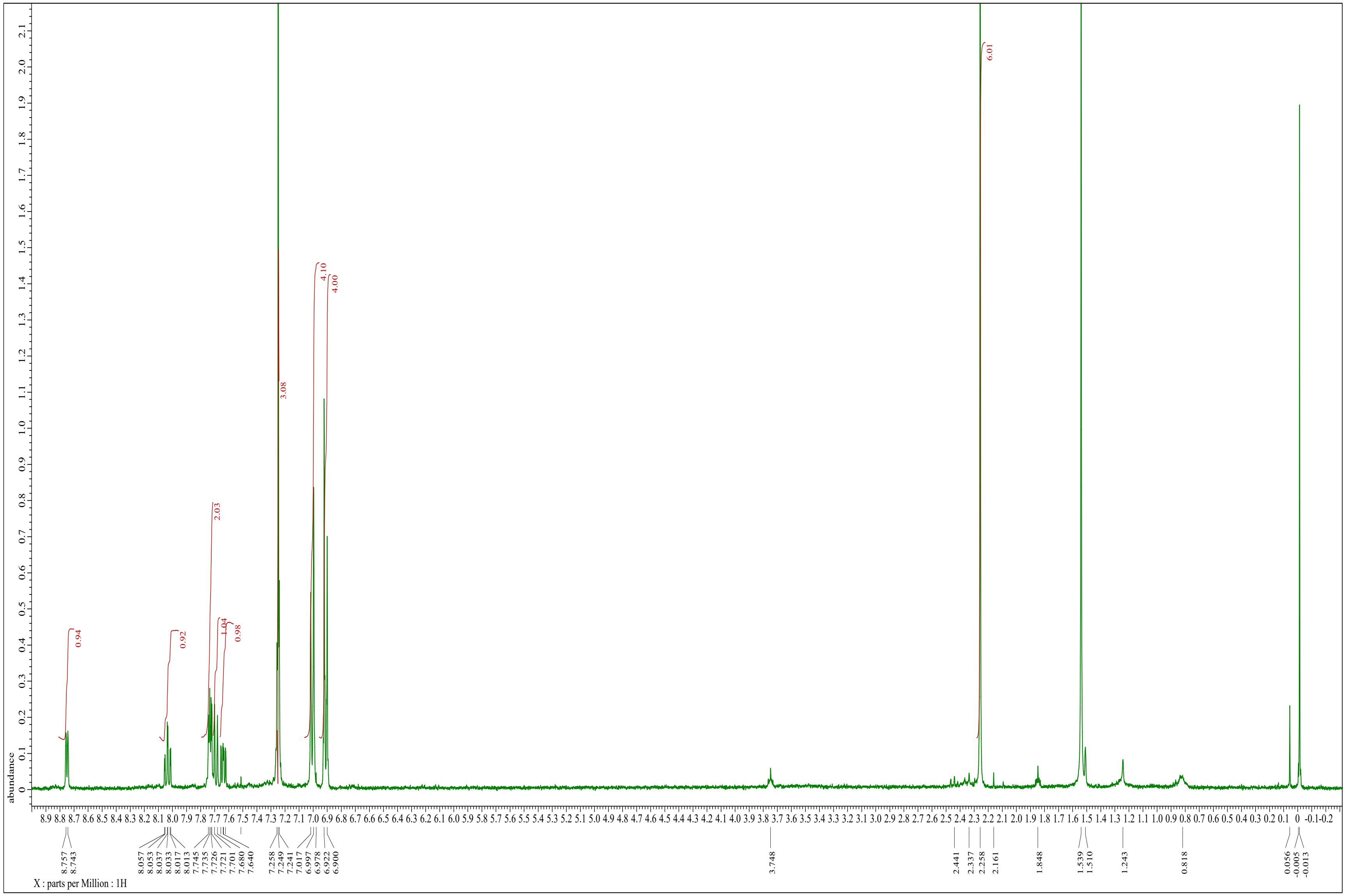

^{13}C NMR charts for 5-*N*-arylaminothiazoles and zinc-thiazole complexes.

5-aminothiazole **4a**

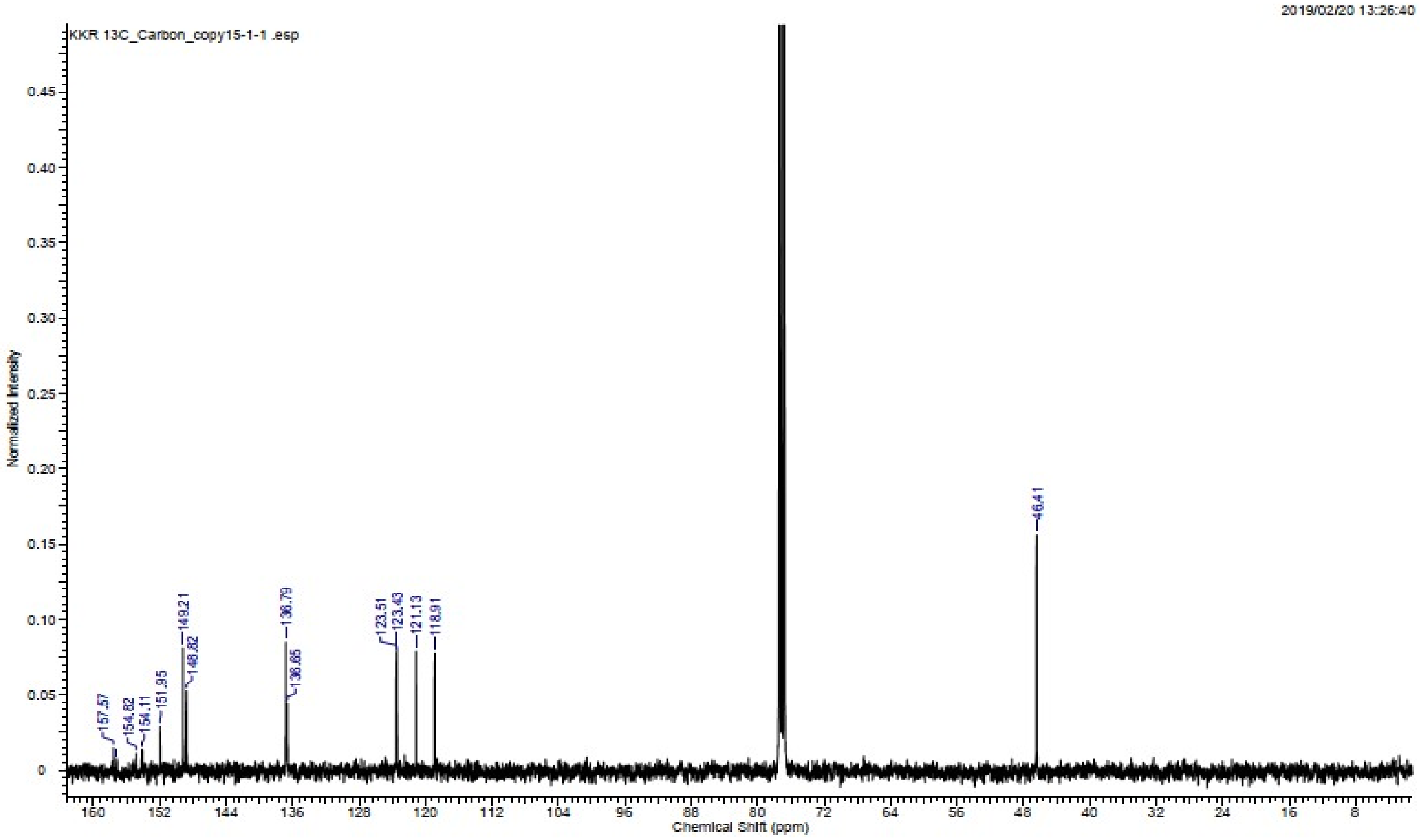

5-aminothiazole **4b**

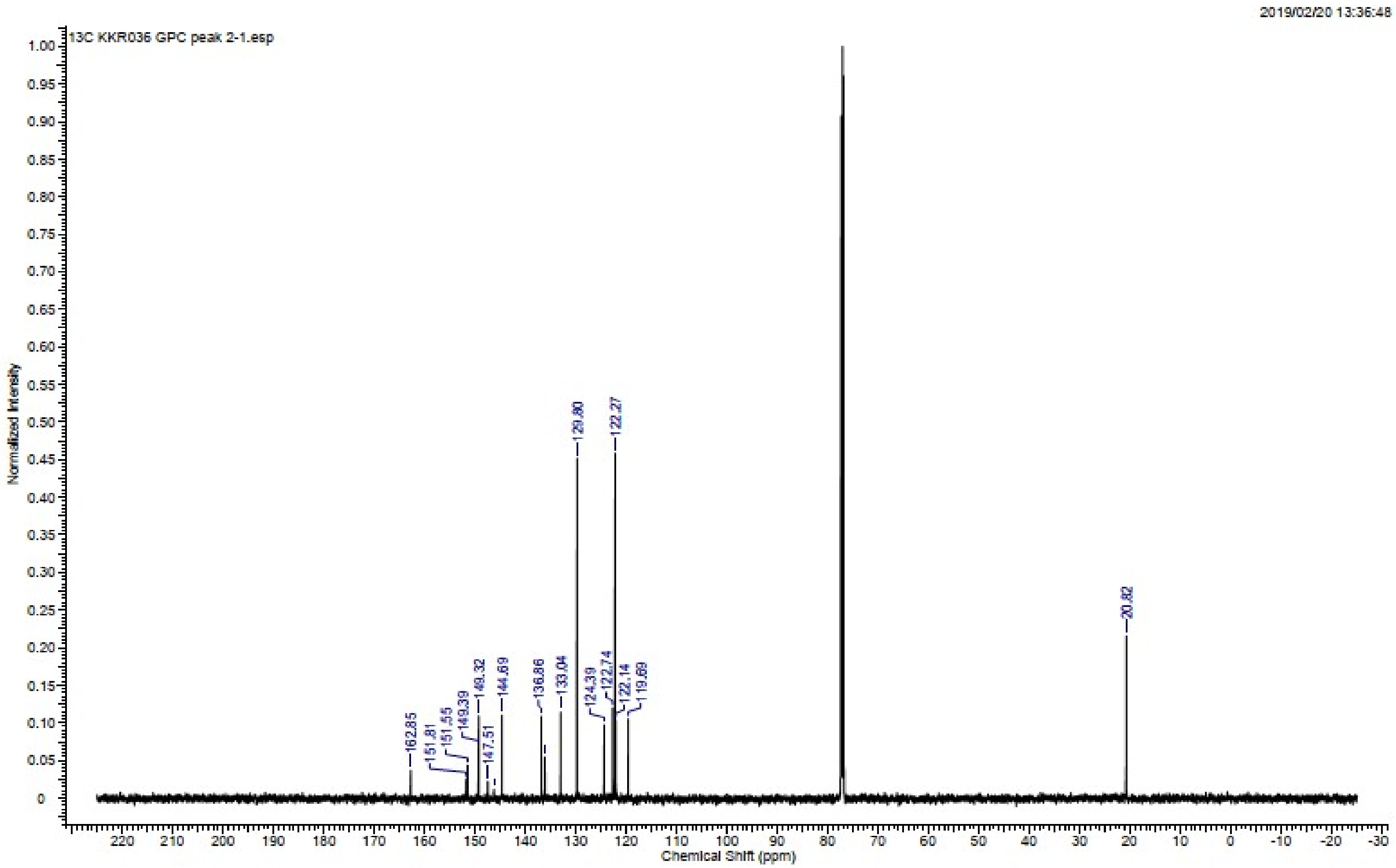

5-aminothiazole **4c**

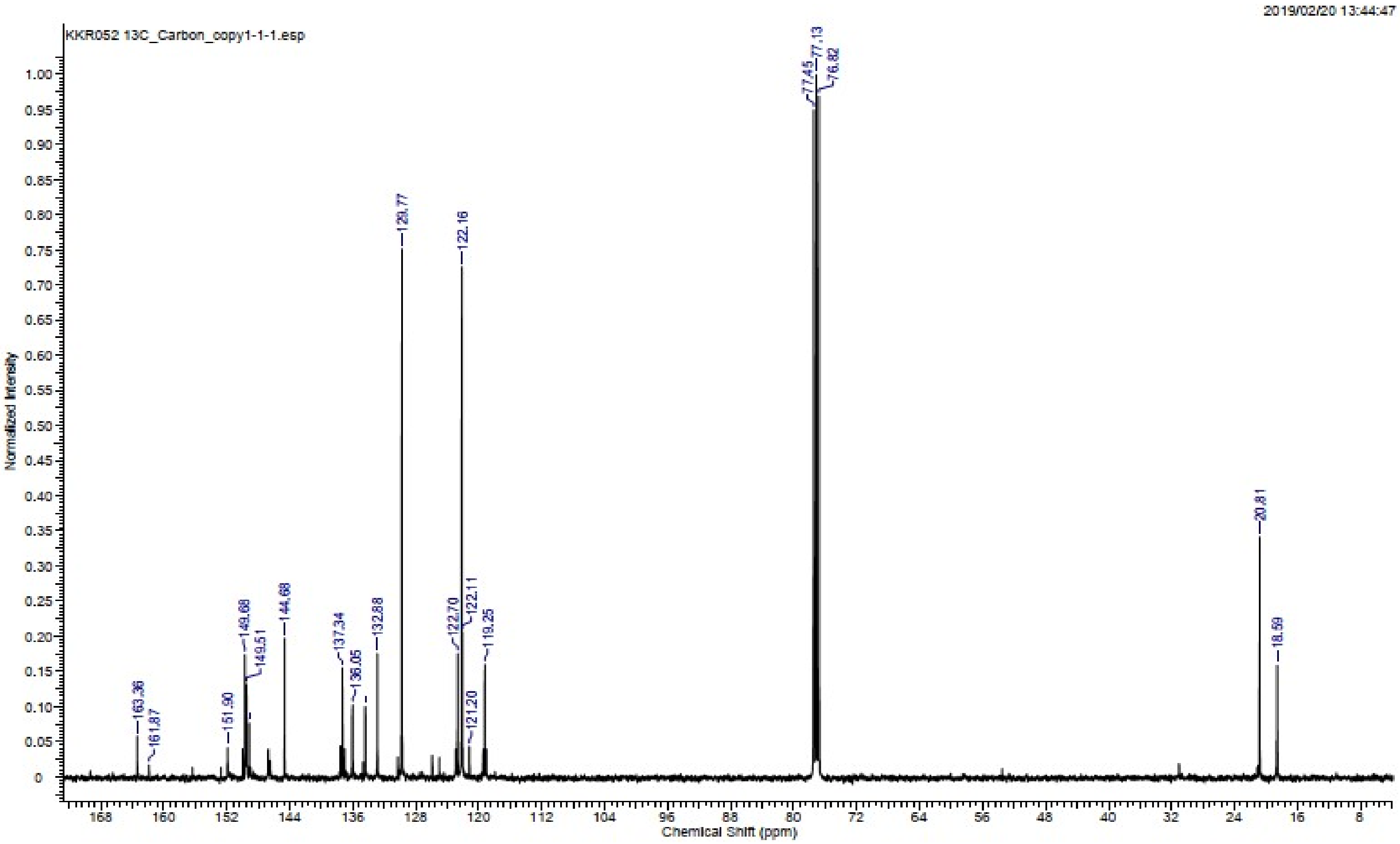

5-aminothiazole **4d**

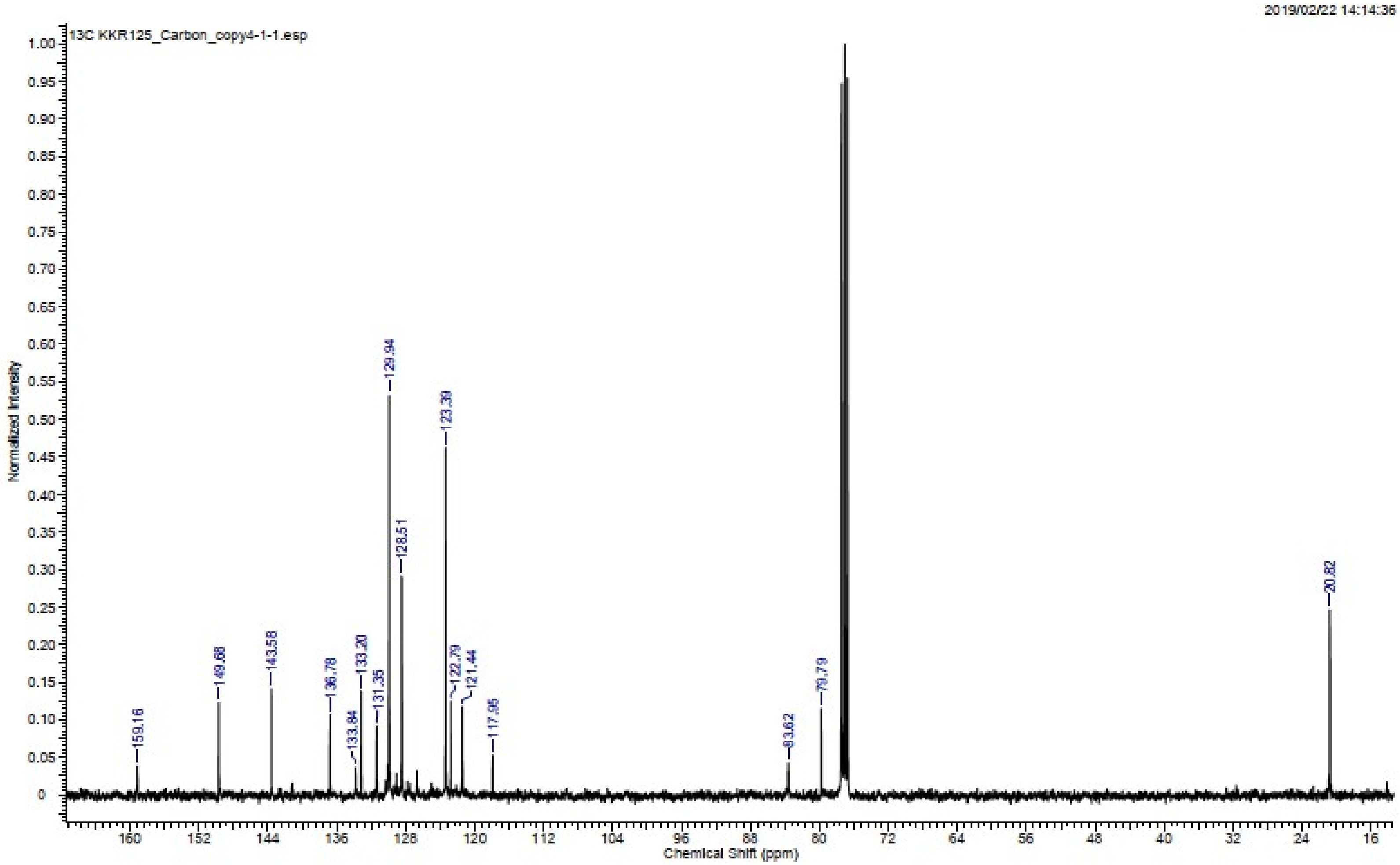

5-aminothiazole **4e**

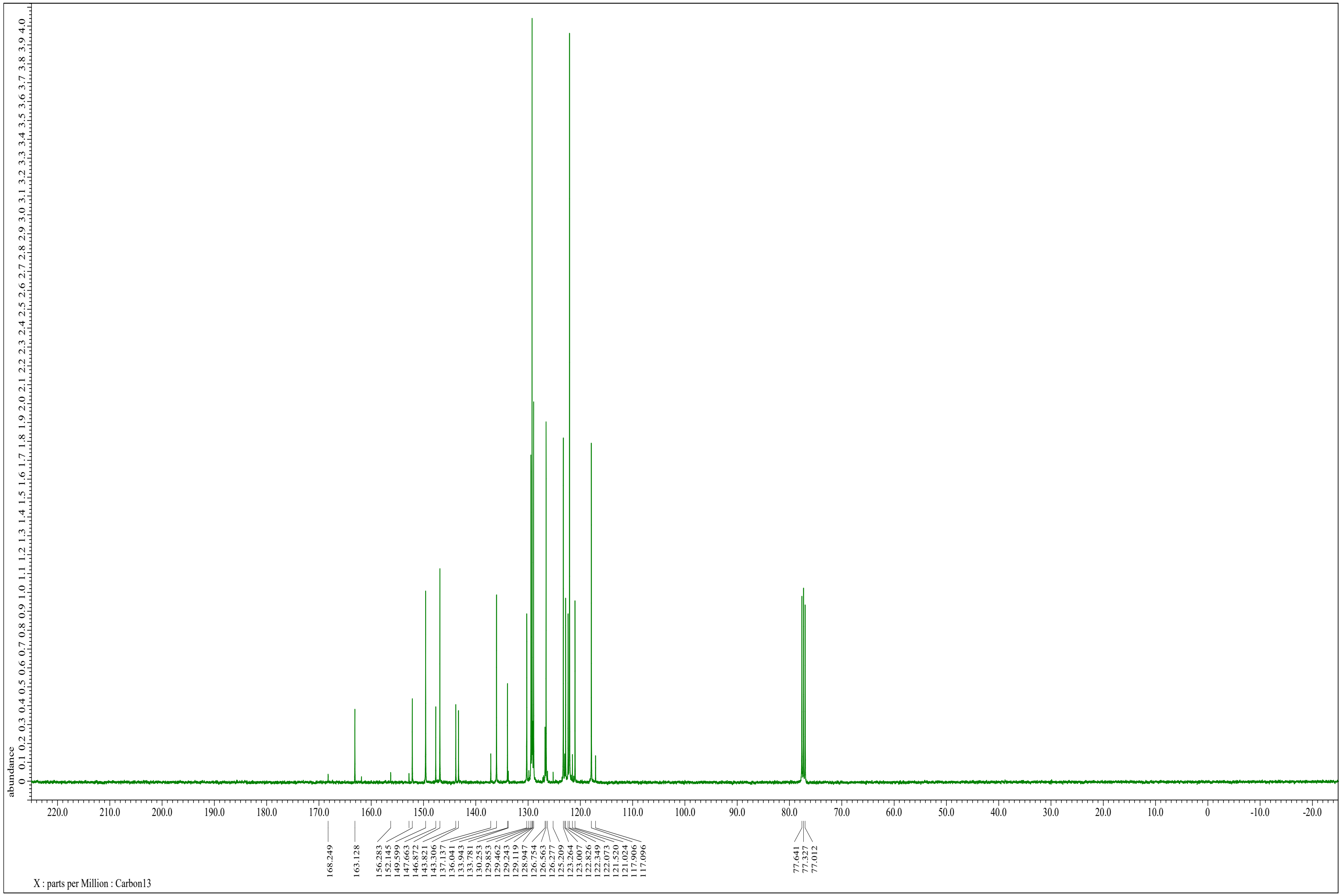

5-aminothiazole **4f**

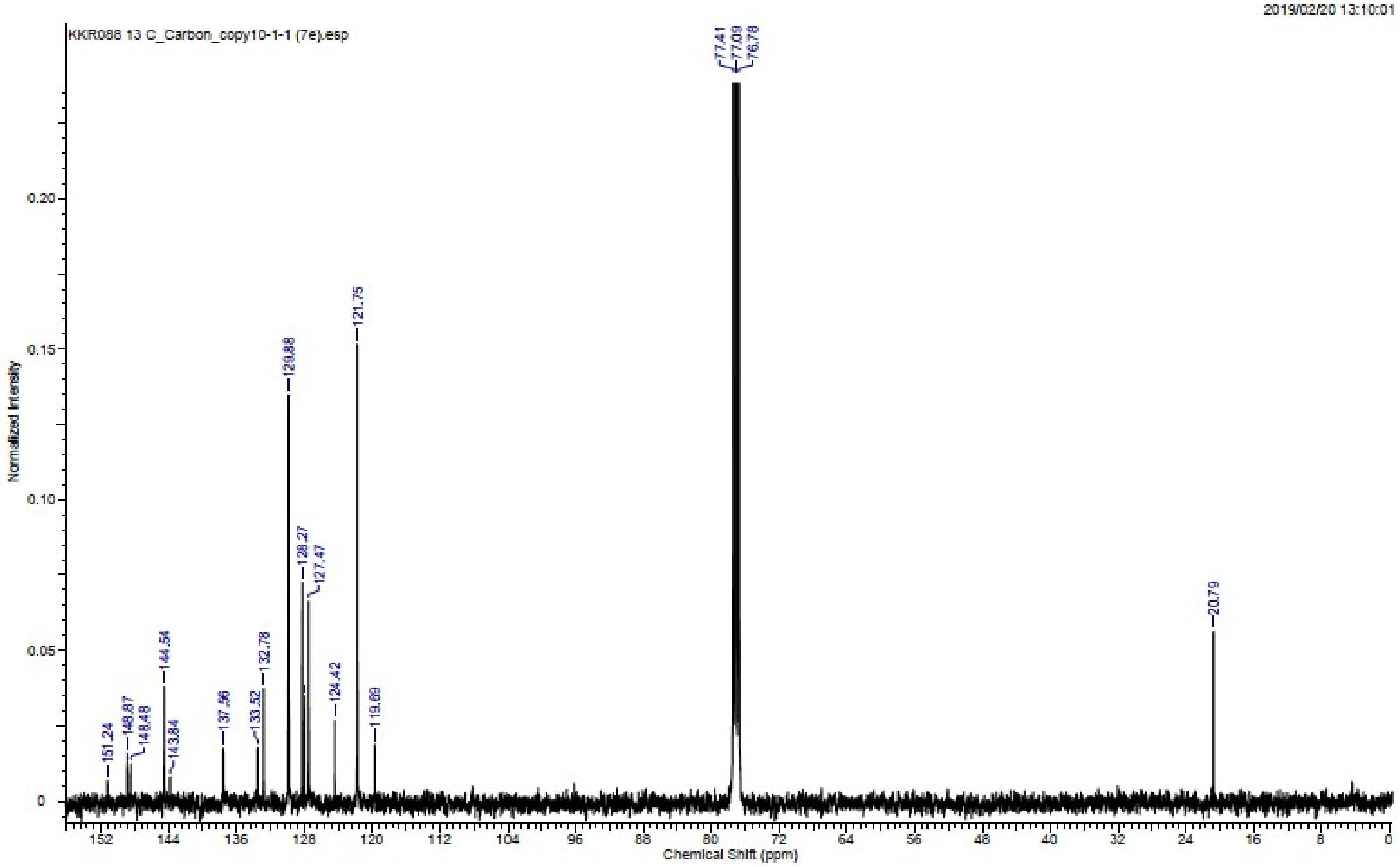

Dipyrromethene type ligand **6a**

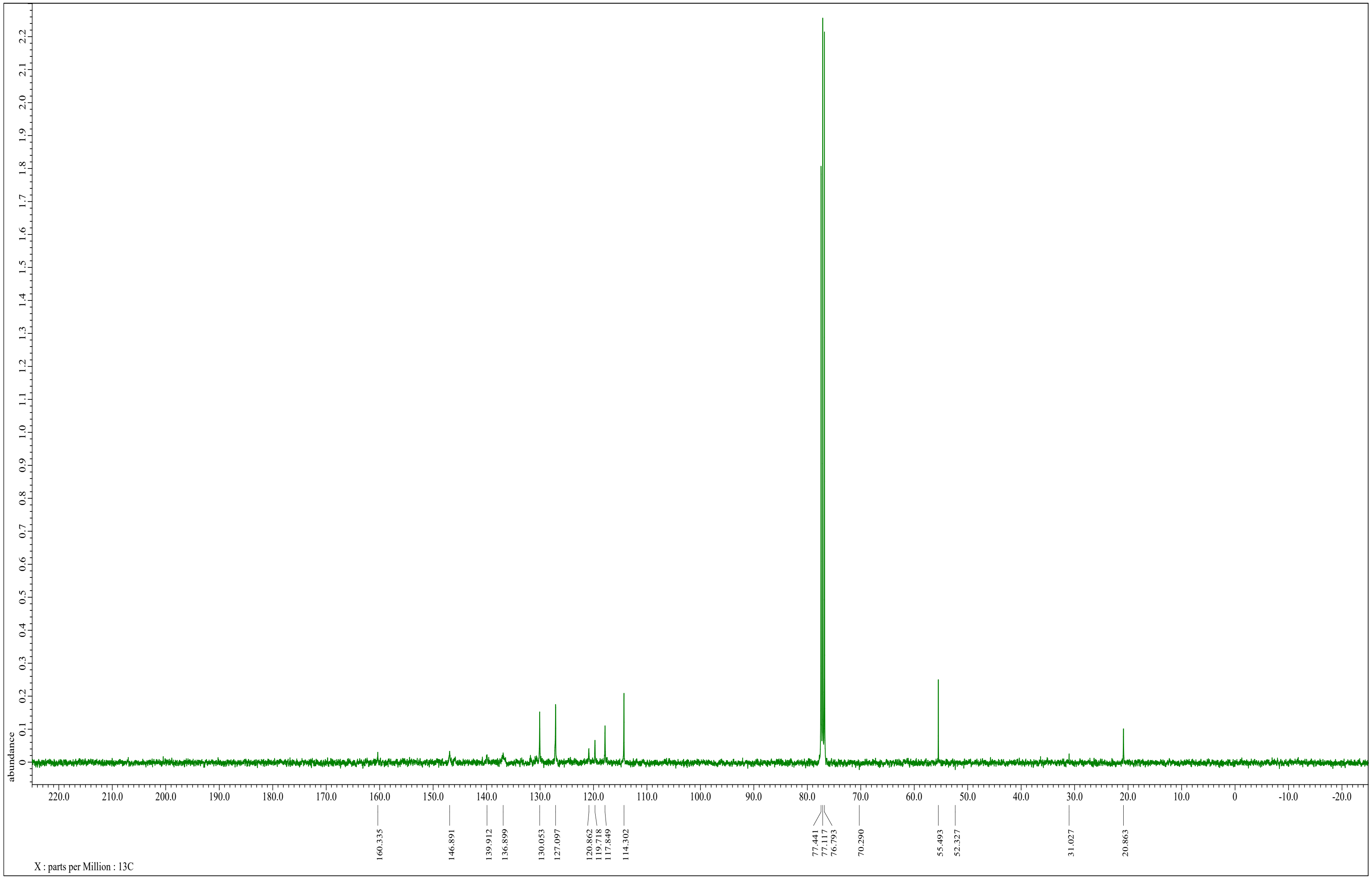
abundance
X : parts per Million : 13C
160.335
146.891
139.912
136.899
130.053
127.097
120.862
119.718
117.849
114.302
77.441
77.117
76.793
70.290
55.493
52.327
31.027
20.863

Dipyrromethene type ligand **6b**

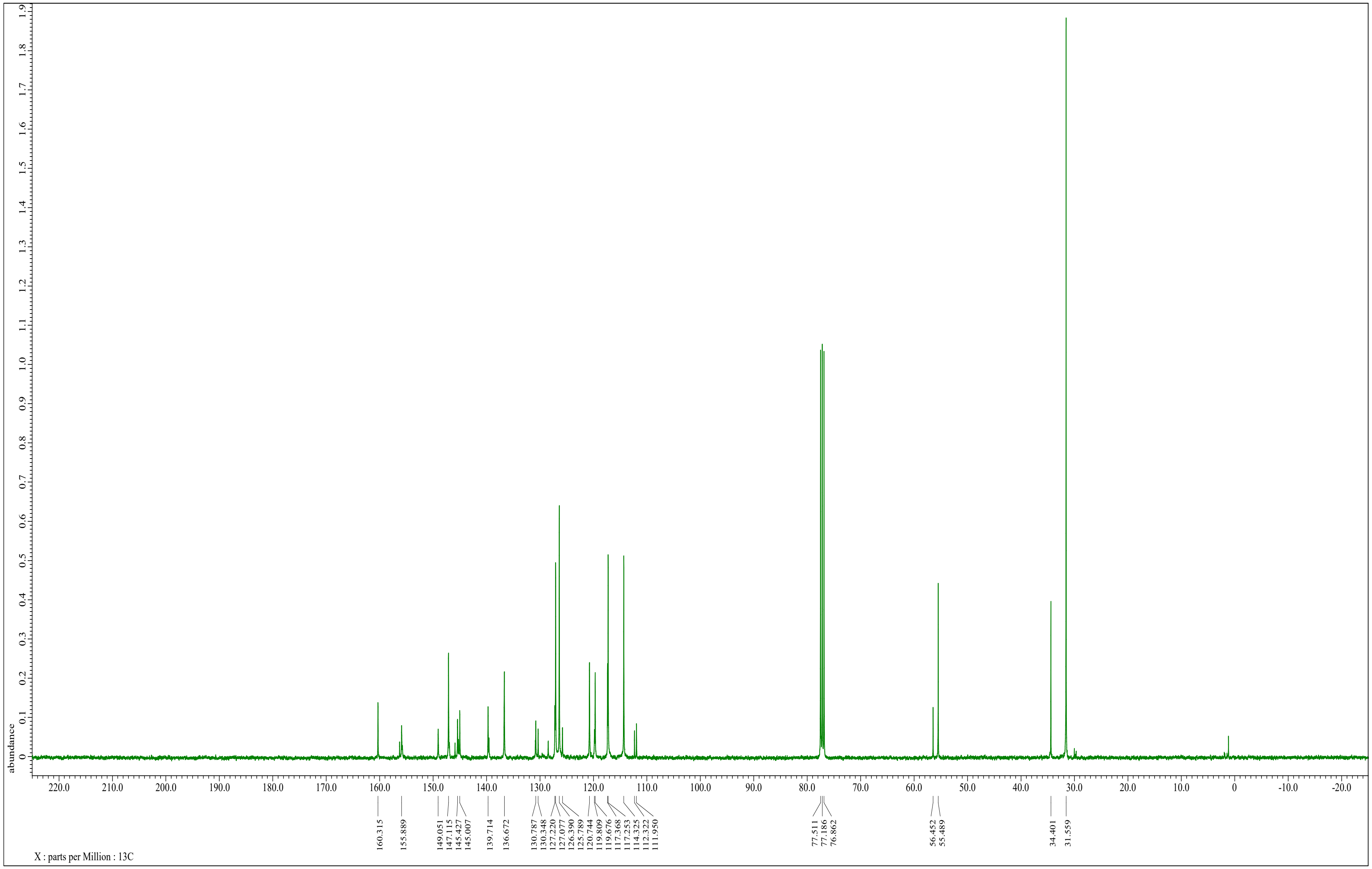

Zinc-thiazole complex **8a**

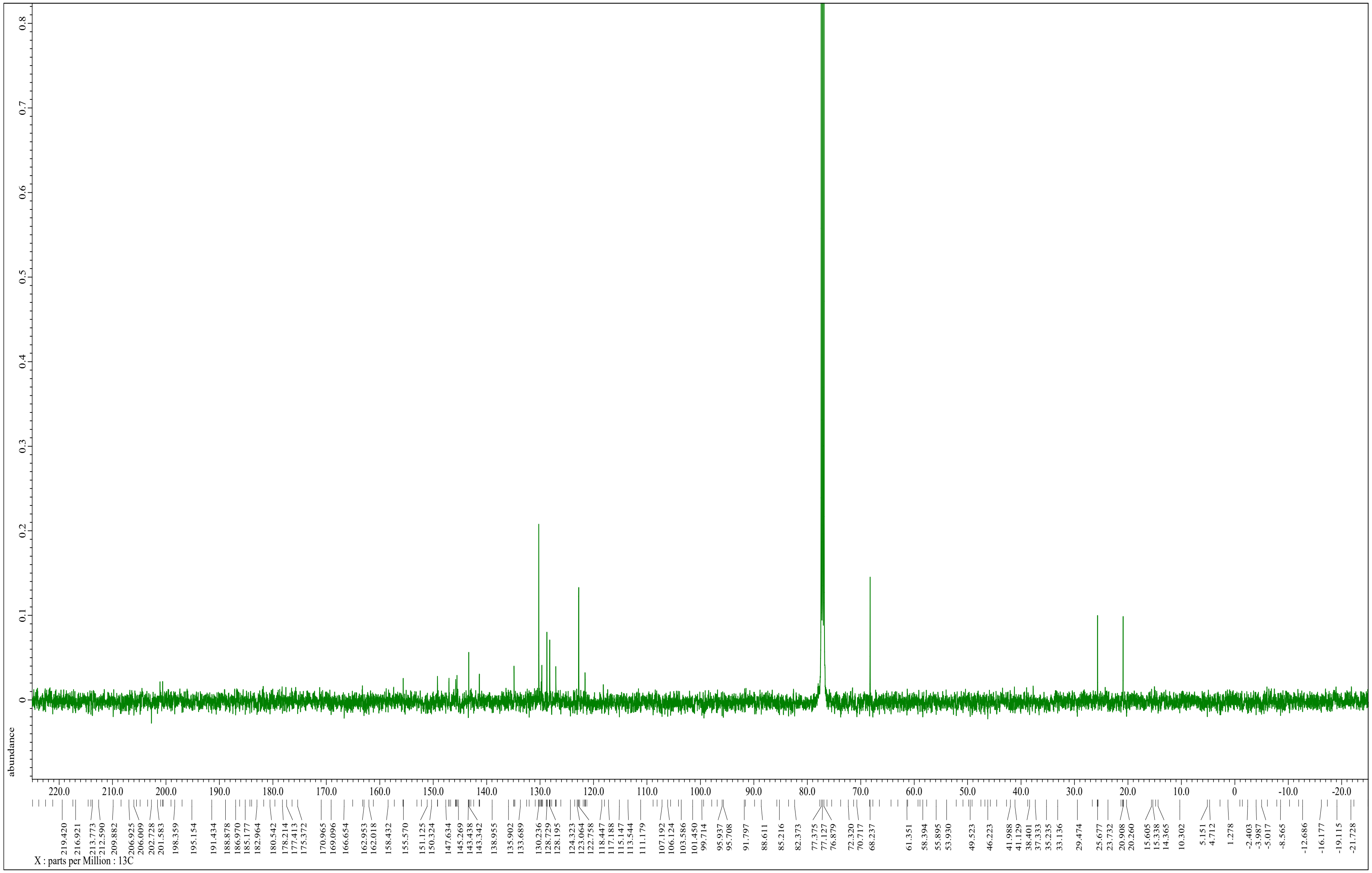

Zinc-thiazole complex **8b**

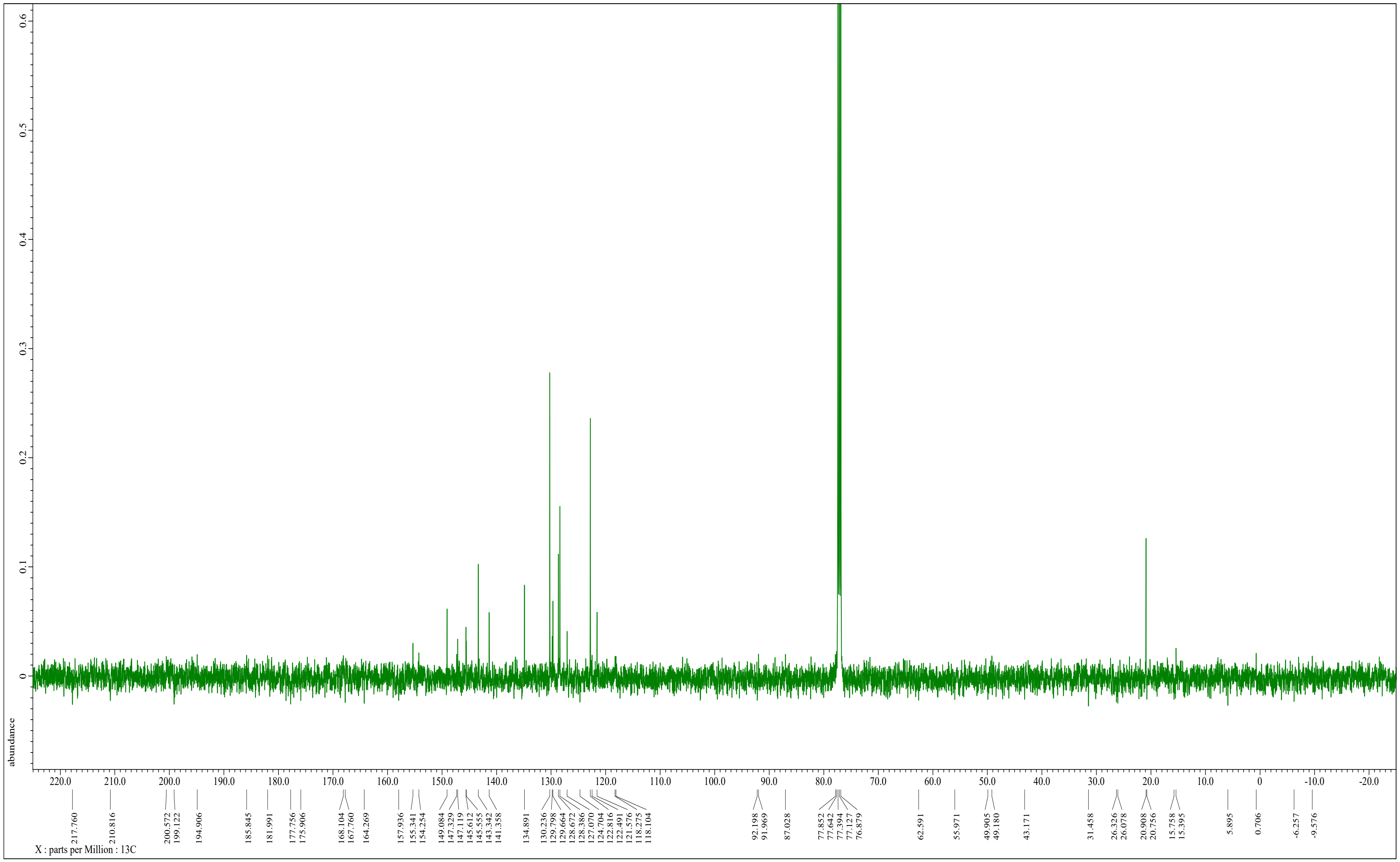

Zinc-thiazole complex **8c**

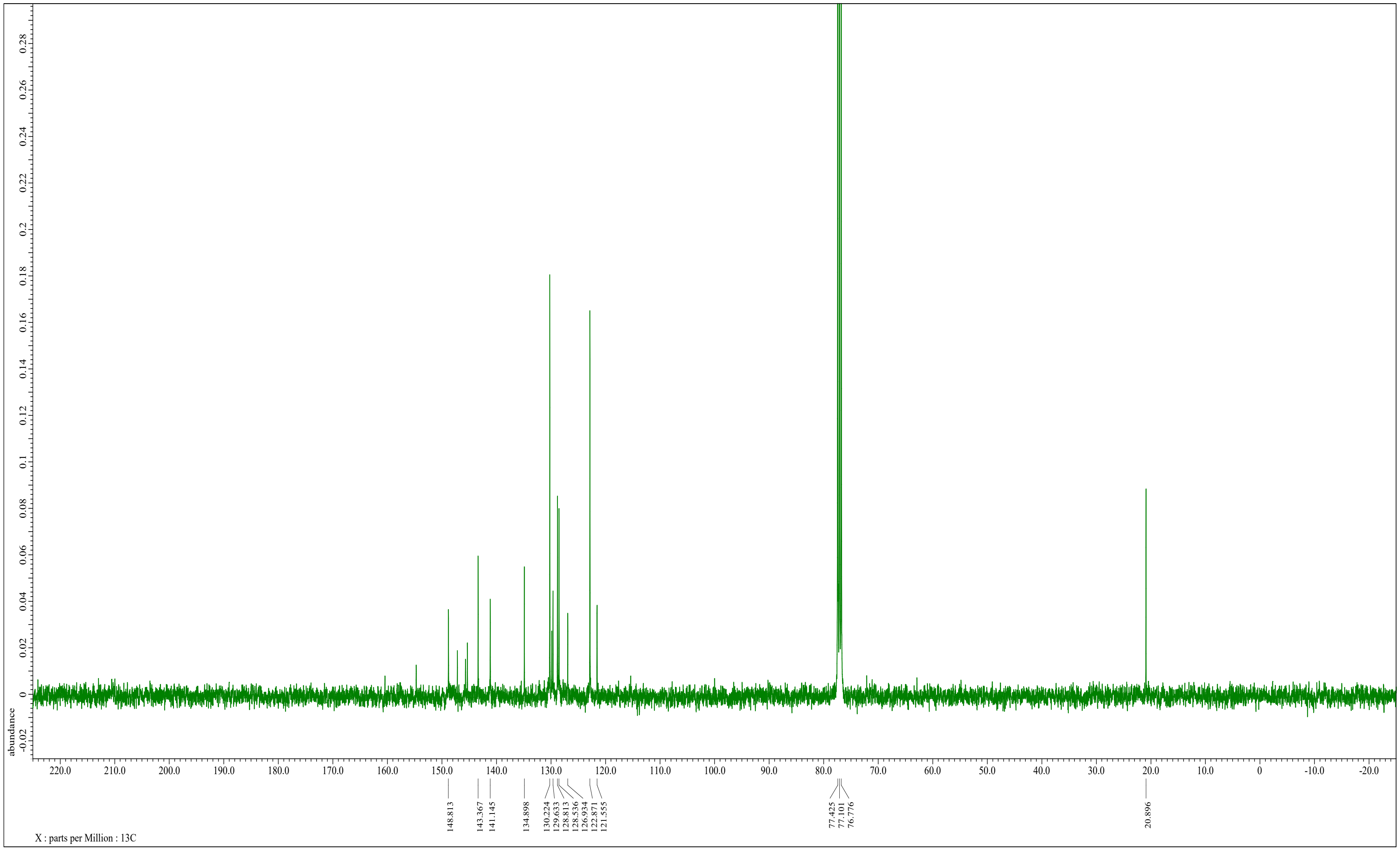

Supplement: RA-012-D2RA01694J-s001 [file RA-012-D2RA01694J-s001.pdf]
